# A global to local genomics analysis of *Clostridioides difficile* ST1/RT027 identifies cryptic transmission events in a northern Arizona healthcare network

**DOI:** 10.1099/mgen.0.000271

**Published:** 2019-05-20

**Authors:** Charles H. D. Williamson, Nathan E. Stone, Amalee E. Nunnally, Heidie M. Hornstra, David M. Wagner, Chandler C. Roe, Adam J. Vazquez, Nivedita Nandurkar, Jacob Vinocur, Joel Terriquez, John Gillece, Jason Travis, Darrin Lemmer, Paul Keim, Jason W. Sahl

**Affiliations:** 1 Pathogen and Microbiome Institute, Northern Arizona University, Flagstaff, AZ 86011, USA; 2 Northern Arizona Healthcare, Flagstaff Medical Center, Flagstaff, AZ 86001, USA; 3 Translational Genomics Research Institute, Flagstaff, AZ 86001, USA

**Keywords:** *Clostridioides difficile*, genomics, transmission, antibiotic resistance

## Abstract

*
Clostridioides difficile* is a ubiquitous, diarrhoeagenic pathogen often associated with healthcare-acquired infections that can cause a range of symptoms from mild, self-limiting disease to toxic megacolon and death. Since the early 2000s, a large proportion of *C. difficile* cases have been attributed to the ribotype 027 (RT027) lineage, which is associated with sequence type 1 (ST1) in the *C. difficile* multilocus sequence typing scheme. The spread of ST1 has been attributed, in part, to resistance to fluoroquinolones used to treat unrelated infections, which creates conditions ideal for *C. difficile* colonization and proliferation. In this study, we analysed 27 isolates from a healthcare network in northern Arizona, USA, and 1352 publicly available ST1 genomes to place locally sampled isolates into a global context. Whole genome, single nucleotide polymorphism analysis demonstrated that at least six separate introductions of ST1 were observed in healthcare facilities in northern Arizona over an 18-month sampling period. A reconstruction of transmission networks identified potential nosocomial transmission of isolates, which were only identified via whole genome sequence analysis. Antibiotic resistance heterogeneity was observed among ST1 genomes, including variability in resistance profiles among locally sampled ST1 isolates. To investigate why ST1 genomes are so common globally and in northern Arizona, we compared all high-quality *C. difficile* genomes and identified that ST1 genomes have gained and lost a number of genomic regions compared to all other *C. difficile* genomes; analyses of other toxigenic *C. difficile* sequence types demonstrate that this loss may be anomalous and could be related to niche specialization. These results suggest that a combination of antimicrobial resistance and gain and loss of specific genes may explain the prominent association of this sequence type with *C. difficile* infection cases worldwide. The degree of genetic variability in ST1 suggests that classifying all ST1 genomes into a quinolone-resistant hypervirulent clone category may not be appropriate. Whole genome sequencing of clinical *C. difficile* isolates provides a high-resolution surveillance strategy for monitoring persistence and transmission of *C. difficile* and for assessing the performance of infection prevention and control strategies.

## Data Summary

Whole genome sequencing data have been deposited in the National Center for Biotechnology Information Sequence Read Archive under BioProject accession number PRJNA438482 (https://www.ncbi.nlm.nih.gov/bioproject/PRJNA438482). Supporting data are provided as supplementary material in figshare: 10.6084/m9.figshare.7966775. The authors confirm all supporting data, code and protocols have been provided within the article or through supplementary data files.

**Impact Statement**

*
Clostridioides difficile* is a major cause of nosocomial infections that can result in toxic megacolon and death. Several lineages of *C. difficile* have been associated with a large proportion of *C. difficile* infections; one of these lineages is labelled as ribotype 027 (RT027) or sequence type 1 (ST1) in the *C. difficile* multilocus sequence typing scheme. Here we analysed RT027/ST1 isolates sampled from clinical settings in northern Arizona. We identified multiple introductions of ST1 into the healthcare network as well as potential nosocomial transmission of closely related isolates. Our results suggest that acquisition of antibiotic resistance and gain and loss of specific genes may explain why the ST1 lineage is routinely associated with *C. difficile* infections. We also introduce an *in silico* ribotyping approach to relate whole genome sequence data to PCR ribotyping information. The current work provides insight into *C. difficile* infections and transmission on a local scale, places local *C. difficile* isolates from a single healthcare network into a global context, and demonstrates the value of incorporating whole genome sequencing and comparative genomics into healthcare surveillance programmes.

## Introduction

*Clostridioides difficile* [1] is one of the most commonly observed diarrhoeal pathogens in hospital settings. *C. difficile* infection (CDI) can range in severity from asymptomatic carriage or mild disease to toxic megacolon and death. The recent rise in the frequency in CDI has been attributed, in part, to the spread of fluoroquinolone-resistant strains of ribotype (RT) 027. Strain CD196, the earliest identified RT027 isolate [2], was isolated in France in 1985 [3]. RT027 was linked to CDI outbreaks in North America and Europe in the 2000s [4–8] and has spread around the world [9]. Although a decrease in the prevalence of RT027 has been reported in some regions [10], the RT027 lineage continues to be routinely isolated from clinical samples [11–16]. Although the mechanisms behind the success of the RT027 lineage are not fully understood, the increase of CDI cases caused by RT027 in the 2000s has been linked to strains with fluoroquinolone resistance [4, 9]. It has also been suggested that the lineage can displace endemic strains [17] and, more recently, Collins and colleagues [18] associated an increased ability to metabolize trehalose with epidemic ribotypes (RT027 as well as RT078), suggesting this trait along with the increased addition of trehalose to foods contributed to the success of RT027.

Many typing methods have been used to characterize *C. difficile* isolated from clinical settings. Ribotyping, a method that relies on differential amplification length profiles of ribosomal intergenic spacer regions, has been one of the most widely used procedures [19, 20]. Multilocus sequence typing (MLST) has also been a commonly applied approach for characterizing *C. difficile* diversity and is more appropriate than ribotyping for examining evolutionary history and relatedness [21]. RT027 is associated with sequence type (ST) 1 in the *C. difficile* MLST scheme [22]. Additional ribotypes associated with ST1 include RT016, RT036 and RT176 [21]. RT016 has been isolated from stool samples associated with CDI in England [23] and RT176 has been associated with CDI in Poland [24] and the Czech Republic [25]. Ribotyping and MLST are increasingly being replaced by comparative genomic methods that rely on whole genome sequence (WGS) data. Ribotyping results, MLST profiles and comparisons utilizing the entire genome are not always congruent [20]; WGS provides the highest resolution for comparative genomics and should be the focus of comparative studies moving forward. The primary focus of this study is a comparison of ST1, which forms a monophyletic clade (see Results) and includes many virulent isolates collected from human clinical samples.

Multiple studies have investigated *C. difficile* with comparative genomics, with several studies focused on disease caused by ST1 (RT027). In one comparative study of three *C. difficile* genomes [2], the authors identified several genomic regions potentially associated with increased virulence of RT027. Since that time, genomic sequences from many additional strains have become available. A large comparative genomics study of *C. difficile* isolates collected from patients with diarrhoea in hospital systems in the UK over a 4-year period indicated that the ST1 lineage was prevalent (17 % of samples) [26]. Another study that included 1290 isolates from patients with CDI in the same area found that 35 % of the isolates were ST1 [27]. He and colleagues [9] investigated the global phylogeny and spread of RT027 (ST1) with whole genome single nucleotide polymorphism (SNP) analysis and identified two epidemic lineages, both of which contained a mutation conferring fluoroquinolone resistance. As these studies demonstrate, ST1 isolates have been frequently identified from clinical samples; other studies have indicated that ST1 isolates are less commonly associated with other environments (soils, dogs, etc.) [28–31]. Although not conclusive, together these studies suggest that ST1 isolates may preferentially colonize the human gut over other environments.

One of the primary concerns about CDI is emerging antimicrobial resistance (AMR). Antibiotic-associated pseudomembranous colitis has been associated with *C. difficile* since the late 1970s [32–34]. Resistance to a variety of antimicrobials has been associated with *C. difficile* isolates, many of which are multi-drug resistant ([35, 36] and references therein). AMR in *C. difficile* may vary between lineages or by region due to differing antimicrobial use and can impact CDI with regard to infection, recurrence and disease outcome [35, 36]. As mentioned previously, fluoroquinolone resistance has been associated with many ST1 (RT027) strains, and this resistance probably contributed, at least partially, to the increase in CDI cases attributed to ST1 during the 2000s as fluoroquinolone use increased during the 1990s and early 2000s [4, 9, 37–39]. Fluoroquinolone resistance in ST1 strains has been associated with mutations in the *gyrA* and *gyrB* genes [40, 41]. Suggested clinical guidelines for treating CDI currently include treatment with vancomycin, fidaxomicin and metronidazole [42]. Although reduced susceptibility to these compounds has been reported, resistance in *C. difficile* has had limited impact on the efficacy of these drugs in the clinic thus far [42, 43]. Understanding AMR in *C. difficile* may yield insights into the prevalence of CDI caused by certain lineages and provide information regarding best practices for prescribing antibiotics.

In this study, we examined 27 ST1 genomes generated as part of a surveillance project across two healthcare facilities in northern Arizona, USA, during 2016 and 2017 and publicly available *C. difficile* genomes. Comparisons were conducted to: (1) characterize northern Arizona ST1 isolates in the context of a worldwide set of genomes; (2) evaluate potential transmission networks within northern Arizona healthcare facilities; (3) use genomics to identify potential mechanisms that have allowed for the widespread presence of the ST1 lineage in northern Arizona and across hospitals worldwide; and (4) better understand the pan-genomics and phylogenomics of the species in general. We also developed an *in silico* ribotyping method for relating whole genome sequence data and ribotyping information.

## Methods

### Genome download and sequence typing

All available *C. difficile* genome assemblies (*n*=1092) were downloaded from GenBank on 30 November 2017. For quality control purposes, statistics were gathered for each genome for number of contigs, number of ambiguous nucleotides (non A,T,G,C) and total genome assembly size. Additionally, all raw sequencing data (paired-end Illumina data only) associated with *C. difficile* available from the Sequence Read Archive (SRA) on 1 September 2017 were downloaded (sratoolkit fastq-dump). For isolates represented by multiple SRA runs, the runs were combined and labelled with the BioSample accession. Data were discarded if the read length was less than 75 bp. The multilocus sequence type (ST) of samples represented by raw read data was determined with stringMLST v0.5 (default settings) [44]. Samples typed as ST1 were assembled with SPAdes v3.10.0 (--careful --cov-cutoff auto -k auto) [45]. These genome assemblies were combined with GenBank assemblies for downstream analyses; in some cases, these reads represent duplicates of GenBank assemblies, but were treated independently in this study due to difficulties with association. Any GenBank assembly or assembly generated with SPAdes that contained more than 1500 contigs, more than 10 ambiguous nucleotides, an anomalous genome assembly size (final data set: <3 600 277 or >4 698 454 bp), or anomalous GC content (final data set: <28.07 or >29.74 mol%) was removed from the data set. In addition, all pairwise mash (v2.0, default sketch size) [46] distances were calculated for all genome assemblies in order to identify genomes annotated as *C. difficile* but belonging to different species. Genomes with an average pairwise mash distance >0.03, which corresponds to <0.97 average nucleotide identity (a conservative cutoff value), were removed from the data set (average mash distances for remaining genomes were less than 0.02). The ST was determined for all assemblies passing quality control metrics with a custom script (https://gist.github.com/jasonsahl/2eedc0ea93f90097890879e56b0c3fa3) that utilizes blastn [47] and the PubMLST database (https://pubmlst.org/) for *C. difficile* [22]. For ST1 genomes, if the ST of the assembly did not match the ST predicted by stringMLST, the assembly was removed from the data set. A total of 1850 genome assemblies from NCBI data (609 GenBank assemblies and 1241 in-house assemblies from the SRA identified as ST1) were included in this study (Table S1, available in the online version of this article). Additionally, publicly available raw sequencing reads representing non-ST1 isolates (*n*=3402) were included in the *in silico* ribotyping analyses.

### *C. difficile* isolation, DNA extraction, sequencing and assembly

Stool samples identified as containing *C. difficile* from two northern Arizona healthcare facilities (labelled facility A and facility B) were collected under IRB No. 764034-NAH and were stored at −80 °C until processing. Isolation of *C. difficile* from stool samples was performed as outlined by Edwards *et al.* [48] with the following modifications; one 10 µl loopful of partially thawed stool was re-suspended in 500 µl of sterile 1× PBS in aerobic conditions. The suspension was immediately transferred to a vinyl Type C anaerobic chamber (Coy Laboratory Products), where 100 µl was plated onto pre-reduced taurocholate-cefotoxin-cyloserine-fructose agar (TCCFA) and incubated at 36 °C for 24–48 h. Suspected *C. difficile* were subcultured onto pre-reduced brain heart infusion agar supplemented with 0.03 % l-cysteine (BHIS) and incubated anaerobically at 36 °C for 24 h. Once isolation of suspected *Clostridioides / Clostridium* species was achieved, a lawn was created (also on BHIS) and incubated anaerobically at 36 °C for an additional 24 h. Genomic DNA was extracted from each isolate, *C. difficile* species identification was confirmed via TaqMan PCR, and all *C. difficile* positive extractions were processed for downstream WGS as described previously [31]. Based on this methodology, a total of 27 ST1 isolates were identified from hospitals in northern Arizona between March 2016 and September 2017 (Table 1). DNA was sequenced on the Illumina MiSeq platform and all genomes were assembled with SPAdes v3.10.0. The average per-contig depth of coverage was calculated with genomeCoverageBed (v2.27.1) [49] from BWA-MEM v0.7.7 alignments [50]. Additionally, 200 bases of each contig was aligned against the GenBank [51] nucleotide database with blastn (v2.7.1) [47] and the identity of the top hit was tabulated. If the alignment was from a known contaminant or from a different species sequenced on the same run, the contig was manually removed from the assembly.

**Table 1.** *
C. difficile* ST1 isolates collected from a healthcare network in northern Arizona

	**Isolation date (YYYY-MM-DD**)	**SRA accession**	**Sequence type**		***In silico*** **genome screen for markers of interest**							
**Isolate name**				**Lineage** ^**1**^	**tcdA/tcdB** ^**2**^	**tcdC** ^**3**^	**cdtA/cdtB** ^**2**^	**gyrA:82** ^**4**^	**TetM** ^**5**^	**ErmB** ^**5**^	**CdeA** ^**5**^	**DfrF** ^**5**^
HS-FS-000016-I-01	2016-04-20	SRR6890740	ST1	FQR1	A+/B+	Identical to CD196	+ / +	I	–	+	+	–
HS-FS-000023-I-01	2016-04-09	SRR6890728	ST1	FQR1	A+/B+	Identical to CD196	+/ +	I	–	+	+	–
HS-FS-000030-I-01	2016-04-05	SRR6890724	ST1	FQR1	A+/B+	Identical to CD196	+/+	I	–	+	+	–
HS-FS-000082-I-02	2016-10-22	SRR6890738	ST1	FQR1	A+/B+	Identical to CD196	+/+	I	+	+	+	+
HS-FS-000084-I-02	2016-10-30	SRR6890739	ST1	FQR1	A+/B+	Identical to CD196	+/+	I	–	+	+	–
HS-FS-000103-I-02	2016-11-15	SRR6890736	ST1	FQR1	A+/B+	Identical to CD196	+/+	I	–	+	+	–
HS-FS-000127-I-03	2016-12-03	SRR6890737	ST1	FQR1	A+/B+	Identical to CD196	+/+	I	–	+	+	–
HS-FS-000148-I-02	2016-12-12	SRR6890734	ST1	FQR1	A+/B+	Identical to CD196	+/+	I	–	+	+	–
HS-FS-000151-I-02	2016-12-08	SRR6890735	ST1	FQR1	A+/B+	Identical to CD196	+/+	I	–	+^6^	+	–
HS-FS-000173-I-03	2017-01-03	SRR6890732	ST1	FQR1	A+/B+	Identical to CD196	+/+	I	–	+	+	–
HS-FS-000020-I-01	2016-04-25	SRR6890741	ST1	FQR2	A+/B+	Identical to CD196	+/+	I	–	–	+	–
HS-FS-000021-I-01	2016-04-27	SRR6890730	ST1	FQR2	A+/B+	Identical to CD196	+/+	I	–	–	+	–
HS-FS-000022-I-01	2016-04-20	SRR6890731	ST1	FQR2	A+/B+	Identical to CD196	+/+	I	–	–	+	–
HS-FS-000024-I-01	2016-04-09	SRR6890729	ST1	FQR2	A+/B+	Identical to CD196	+/+	I	–	–	+	–
HS-FS-000025-I-01	2016-03-29	SRR6890726	ST1	FQR2	A+/B+	Identical to CD196	+/+	I	–	–	+	–
HS-FS-000031-I-01	2016-03-26	SRR6890725	ST1	FQR2	A+/B+	Identical to CD196	+/+	I	–	–	+	–
HS-FS-000034-I-02	2016-05-04	SRR6890722	ST1	FQR2	A+/B+	Identical to CD196	+/+	I	–	–	+	–
HS-FS-000043-I-01	2016-05-31	SRR6890723	ST1	FQR2	A+/B+	Identical to CD196	+/+	I	–	–	+	–
HS-FS-000057-I-02	2016-07-09	SRR6890747	ST1	FQR2	A+/B+	Identical to CD196	+/+	I	–	–	+	–
HS-FS-000091-I-02	2016-09-16	SRR6890746	ST1	FQR2	A+/B+	Identical to CD196	+/+	I	–	–	+	–
HS-FS-000188-I-02	2017-01-11	SRR6890733	ST1	FQR2	A+/B+	Identical to CD196	+/+	I	–	–	+	–
HS-FS-000251-I-02	2017-05-30	SRR6890745	ST1	FQR2	A+/B+	Identical to CD196	+/+	I	–	–	+	–
HS-FS-000027-I-01	2016-04-29	SRR6890727	ST1		A+/B+	Identical to CD196	+/+	T	–	–	+	–
HS-FS-000194-I-03	2017-02-05	SRR8166226	ST1		A+/B+	Identical to CD196	+/+	T	–	–	+	–
HS-FS-000264-I-02	2017-07-29	SRR6890744	ST1		A+/B+	Identical to CD196	+/+	T	–	–	+	–
HS-FS-000287-I-02	2017-08-30	SRR6890743	ST1		A+/B+	Identical to CD196	+/+	T	–	–	+	–
HS-FS-000292-I-03	2017-09-06	SRR6890742	ST1		A+/B+	Identical to CD196	+/+	T	–	–	+	–

1, Lineages defined in He *et al.* 2013. 2, present if BSR value >0.8. 3, BSR value=1. 4, GyrA mutation inferring fluoroquinolone resistance: I=resistant and T=susceptible. 5, present if BSR value >0.9. 6, two putative copies.

### Simulated reads

Genome assemblies have been shown to provide more noise when inferring the phylogenetic structure of a species compared to raw read data [52]. For all publicly available genome assemblies downloaded from GenBank and passing filters, simulated reads were generated with ART [53] version MountRainier with the following command: art_illumina -ss MSv3 -l 250 f 75 m 300 s 30. Simulated reads instead of genome assemblies were used for SNP discovery and phylogenetics for ST1 genomes.

### Phylogenetic analyses

To generate a preliminary phylogeny, genome assemblies (*n*=1877) were aligned against the finished genome of *C. difficile* CD630 (GCA_000009205.1) with NUCmer (MUMmer v3.23) [54], and SNPs were called in conjunction with NASP v1.1.2 [52]. A maximum-likelihood phylogeny was inferred on an alignment of 85 331 concatenated SNPs (Table S2) called from a core genome alignment of 1 860 526 positions with IQ-TREE v1.6.1 [55] using the best-fit model (GTR+F+ASC+R5) identified by ModelFinder [56] and the UFBoot2 ultrafast bootstrapping option [57]. Phangorn v.2.4.0 [58] was used to calculate the consistency index (excluding parsimony-uninformative SNPs) and the retention index (using all SNPs) [59]. All trees were visualized in FigTree v1.4.3 (http://tree.bio.ed.ac.uk/software/figtree/) or the Interactive Tree of Life online tool [60].

To generate a phylogeny for ST1 genomes (*n*=1379), short read data (simulated reads for genome assemblies downloaded from NCBI and actual Illumina short reads for publicly available data and 27 newly sequenced genomes) were aligned against the finished ST1 genome CD196 (GCA_000085225.1) with BWA-MEM v0.7.7 [50]. SNPs were called from the alignments with the Unified Genotyper method in GATK v3.3–0 [61, 62]. Positions in duplicated regions of the reference genome (identified with NUCmer), positions with less than 10× coverage, and positions with a mixture of alleles (<0.9 single allele) were removed from the analyses. SNP positions with calls in at least 90 % of the analysed genomes were concatenated (*n*=4283 SNPs, Table S3). A maximum-likelihood tree was inferred with IQ-TREE v1.6.1 (best-fit model TVMe+ASC+R2). The tree was rooted with the ST1 genome ERR030325 based upon previous phylogenetic analysis. Phangorn was used to calculate the consistency index and retention index as described above.

### Root-to-tip regression and divergence time analysis

To estimate the date of divergence of the successful ST1 lineage, a set of genomes (*n*=90; 87 ST1 genomes and three outgroup genomes) for which accurate sample dates were known were analysed. SNPs were called with NASP [52] and the program PHIPack [63] was used to test for evidence of recombination, as this can confound divergence-dating analyses. To calculate accurate divergence times, recombination in ST1 SNPs was identified and removed using the program ClonalFrameML [64]. Non-recombinatory SNP positions present in all ST1 genomes were processed further (Table S4). The presence of a temporal signal was assessed through regression analysis implementing root-to-tip genetic distance as a function of the sample year in the program TempEst version 1.5.1 [65] (http://tree.bio.ed.ac.uk/software/tempest/). The determination coefficient, *R* ^2^, was used as a measure of clocklike behaviour with the best-fitting root selected in an effort to maximize *R* ^2^. Additionally, 10 000 random permutations of the sampling dates over the sequences were performed in an effort to evaluate the significance of the regression results [66].

The best nucleotide substitution model was inferred using the Bayesian information criterion in the software IQ-TREE version 1.5.5 [55]. beast version 1.8.4 [67]was used to estimate evolutionary rates and time to the most recent common ancestor (TMRCA) through a Bayesian molecular clock analysis using tip dating. beast analysis was run with a correction for invariant sites by specifying a Constant Patterns model in the beast xml file. The numbers of constant As, Cs, Ts and Gs were added to the beast xml file. A ‘path and stepping stone’ sampling marginal-likelihood estimator was used to determine the best-fitting clock and demographic model combinations [68]. The log marginal likelihood was used to assess the statistical fits of different clock and demographic model combinations (Table S5). Four independent chains of one billion iterations were run for the best clock and demographic model combination. Convergence among the four chains was confirmed in the program Tracer version 1.6.0 (http://tree.bio.ed.ac.uk/software/tracer/).

### *C. difficile* transmission and persistence analysis.

To provide insight into the persistence and transmission of *C. difficile* within and among healthcare facilities in northern Arizona, whole genome SNP phylogenies were coupled with epidemiological data. For northern Arizona isolate genomes within lineages of interest, core genome SNPs were called with NASP (reference CD196; GCA_000085225.1) and maximum-parsimony phylogenies were inferred with Phangorn [58] (parsimony trees were used for easily mapping the number of SNP differences between isolates). Epidemiological data were collected from healthcare facilities but, in some cases, information was incomplete.

### *In silico* predicted AMR profiling

Proteins (*n*=2177) from the comprehensive antimicrobial resistance database (CARD) [69] were downloaded on 18 December 2017. Protein sequences were aligned against all ST1 genomes with the tblastn option in LS-BSR. Any peptide with a blast score ratio (BSR) [70] >0.9, which is equivalent to 90 % identity over 100 % of the peptide length [71], was investigated further. As antibiotic resistance within *C. difficile* has been associated with mobile genetic elements [72, 73], the ST1 genomes were screened for the presence of a set of transposons: Tn916 (accession U09422), Tn1549 (AF192329), Tn4451 (U15027), Tn4453a (AF226276), Tn5397 (AF333235), Tn5398 (AF109075), Tn6194 (HG475346), Tn6215 (KC166248), Tn6218 (HG002387), TnB1230 (AJ222769), CTn1-like (extracted from *C. difficile* R20291 genome FN545816.1: 4099441–4125374) [72], Tn6192 (FN545816.1: 2087833–2125356), and Tn6105 (FN545816.1: 2059876–2074593). Sequence reads were aligned to the reference transposon sequences with BWA-MEM v0.7.7 [50] and the breadth of coverage for analysed transposon sequences (depth of coverage of 3×) was used as an indicator of presence or absence of the transposon. blastn and tblastn [47] were also used to test for the presence of a transposon.

Several mutations associated with fluoroquinolone resistance in *C. difficile* have been published in the literature [36, 40, 41, 74]. For GyrA (CD630DERM_00060) and GyrB (CD630DERM_00050), predicted protein sequences were extracted for all genome assemblies from tblastn alignments and then aligned with muscle v3.8.31 [75]. The states of mutations associated with fluoroquinolone resistance were manually investigated.

### Antibiotic resistance testing

Four ST1 isolates collected as part of this study (*n*=27) exhibiting various *in silico* predicted AMR profiles were screened for resistance to vancomycin, tetracycline and ciprofloxacin on Brucella blood agar using Etests (bioMérieux). These isolates were chosen as they represented the diversity of *in silico* predicted AMR profiles among northern Arizona isolates. Inhibition ellipses were examined at 24 and 48 h. Minimum inhibitory concentration (MIC) breakpoints were based upon recommendations by CLSI (https://clsi.org/), the European Committee on Antimicrobial Resistance Testing (http://www.eucast.org/clinical_breakpoints/) and the available literature [41] and are as follows: vancomycin: <2 µg ml^−1^ susceptible, 2–4 µg ml^−1^ intermediate, >4 µg ml^−1^ resistant; tetracycline: >16 µg ml^−1^ resistant; ciprofloxacin: >16 µg ml^−1^ resistant.

### Comparative genomics

For large-scale comparative genomics, genome assemblies were processed with the LS-BSR pipeline [76]. In each genome coding regions (CDSs) were predicted with Prodigal v2.60 [77] and clustered with USEARCH v10.0.240_i86linux32 [78] at an identity of 0.9. A representative sequence for each cluster was then aligned against all analysed genomes with BLAT v35x.1 [79]. Scripts provided with the LS-BSR tool were used to identify core genome CDSs (pan_genome_stats.py) and compare BSR values of CDSs among groups of genomes (compare_BSR.py). To identify CDSs potentially differentially conserved among groups of interest, BSR values for individual CDSs were compared between genomes belonging to different STs (ST1: *n*=1379; ST8: *n*=31; ST15: *n*=34; and ST63: *n*=13). A CDS was considered conserved in a group (ST) if the BSR value for the CDS was greater than 0.8 for greater than 95 % of target-group genomes and the BSR value was greater than 0.4 in less than 5 % of genomes of the non-target group. To complement the LS-BSR pipeline and account for fragmented draft assemblies, CDS/region presence/absence was also analysed by aligning sequence reads to CDSs/regions/genomes of interest with BWA-MEM and evaluating the presence of a CDS/region in a genome based upon breadth of coverage (>80 %) at a depth of coverage of 3×.

Gene regions have been identified in ST1 genomes that have been linked to virulence [2] (Table S6). Because these regions were discovered utilizing a small number of genomes, we screened the peptide sequences from these regions against all genomes with LS-BSR using the tblastn alignment option. Additionally, genome assemblies were screened for genomic features associated with trehalose metabolism [18] (Table S6). For TreR (CBA65726.1), sequences were extracted for all genome assemblies from tblastn alignments and then aligned with muscle [75]. The state of a mutation associated with increased trehalose metabolism was manually investigated. Genome assemblies were also screened for proteins encoded by a four-gene region associated with increased trehalose metabolism (FN665653.1: 3231100–3237100) with LS-BSR using tblastn.****

### *In silico* ribotyping

Standard ribotyping primers (5′-GTGCGGCTGGATCACCTCCT-3′, 5′-CCCTGCACCCTTAATAACTTGACC-3′) [19] were aligned against all completed ST1 genomes with an *in silico* PCR script (https://github.com/TGenNorth/vipr) and the predicted amplicon products were identified. Seven amplicons of defined lengths (Table S7) were conserved across all completed ST1 genomes. To test the ability of these amplicons to differentiate genomes, raw sequencing reads representing 4643 genomes (initial test set data downloaded from SRA in September 2017) were mapped against these seven amplicons with Kallisto [80] using the ‘--bias’ correction. For each sample, an amplicon was determined to be present if the read count for that amplicon was at least 20 % of the maximum read count for any of the seven amplicons for that sample; this allows for a small amount of indiscriminate read counting. The PCR ribotype for many of these samples was unknown; therefore, to further test the *in silico* ribotyping approach, sequencing reads representing 624 *C* *. difficile* isolates that have been PCR ribotyped as part of another study [81] were also evaluated. This data set included multiple ribotypes (RT027 and RT176) within the ST1 lineage [81]. The *in silico* ribotyping approach was also applied to northern Arizona isolates (this study, *n*=27).

For comparison with *in silico* results, PCR ribotyping was performed for 10 *C* *. difficile* samples representing multiple sequence types. Ribotyping PCRs were conducted using the same forward and reverse primers described by Janezic *et al.* 2011 [82]. PCRs were carried out in 50 µl volumes containing the following reagents (given in final concentrations): 5–10 ng of gDNA template, 1× PCR buffer, 1.5 mm MgCl_2_, 0.2 mm dNTPs, 0.1 mg ml^−1^ BSA, 1.25 U Platinum *Taq* polymerase, and 1.0 µm of each primer. PCRs were cycled according to the following conditions: 95 °C for 5 min to release the polymerase antibody, followed by 35 cycles of 95 °C for 60 s, 57 °C for 30 s and 72 °C for 60 s, followed by a final elongation step of 72 °C for 10 min. We then conducted a PCR purification step aimed at removing primer dimer and unincorporated dNTPs using Agencourt AMPure XP beads (Beckman Coulter) at a 1 : 1 ratio (50 µl PCR product to 50 µl beads) according to the manufacturer’s recommendations with the following modifications: wash steps were conducted using 80 % ethanol and final products were eluted in 25 µl of 0.01 m Tris-HCl buffer, supplemented with 0.05 % Tween20. Electropherograms were generated for each of the PCR products using an Agilent 2100 Bioanalyzer (Agilent Technologies, Catalogue no. G2939BA), which provides greater resolution and specificity than a standard agarose gel. Specifically, the Agilent DNA 1000 kit (Agilent Biotechnologies, Catalogue no. 5067–1504) was used to analyse the sizing, quantity and separation patterns of 1 µl for each of the DNA libraries.

### Genome accession information

ST1 genomes for isolates collected from healthcare facilities in northern Arizona were deposited in the National Center for Biotechnology Information Sequence Read Archive under BioProject accession PRJNA438482 (https://www.ncbi.nlm.nih.gov/bioproject/PRJNA438482). Individual accession numbers are shown in Table 1.

## Results

*C. difficile* ST1 isolates from two healthcare facilities in northern Arizona were examined to understand the diversity of ST1 strains circulating in northern Arizona in the context of a global collection of *C. difficile *, to identify potential transmission events within a single healthcare network, and to characterize AMR and the core/pan-genome within the ST1 lineage.

### Phylogenetic diversity of *C. difficile*

**Fig. 1.**
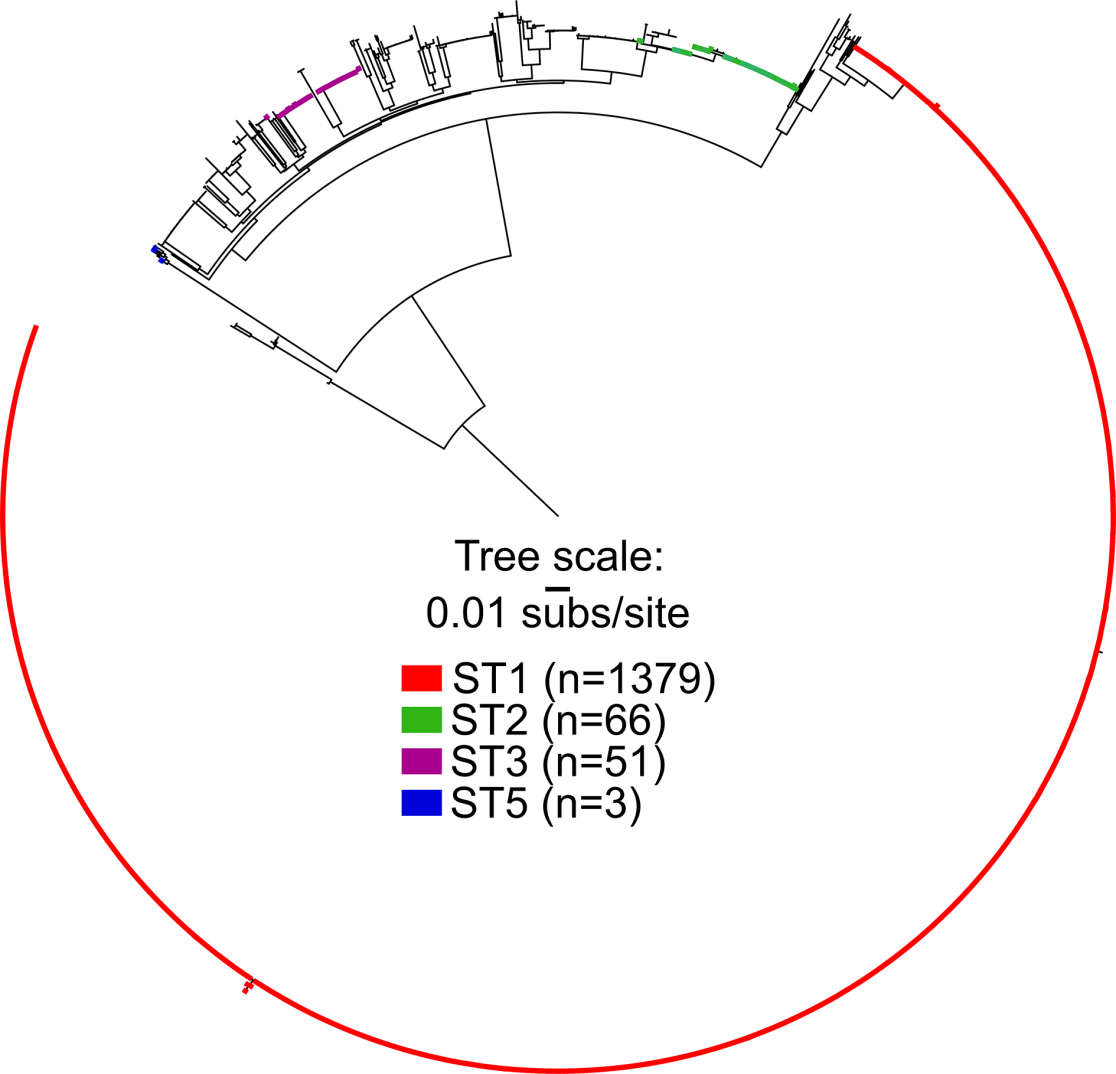
Maximum-likelihood phylogeny of a global collection of *C. difficile* genomes (*n*=1877) inferred from 85 331 SNPs showing the position of ST1 as well as other STs. ST1 forms a monophyletic clade (red) whereas other STs are paraphyletic (green, purple, blue).

A maximum-likelihood phylogeny (Fig. 1) was inferred on a concatenation of 85 331 SNPs (Table S2) identified from a 1 860 526 nt core genome alignment; this analysis included all *C. difficile* genome assemblies that passed through all quality filters (*n*=1877; including 1379 ST1 genomes). The consistency index (excluding parsimony-uninformative SNPs) of this phylogeny was 0.30 and the retention index was 0.97. It is important to note that not all currently described STs are represented by genome assemblies included in this analysis. For example, ST11 genome assemblies, which include RT078, were not included as they were filtered out based on large mash distances. The results demonstrate that genomes identified as ST1, including all 27 isolates from northern Arizona, form a monophyletic clade. Other sequence types, such as ST2, ST3 and ST5, are paraphyletic, demonstrating the limitations of using sub-genomic sequence typing information to infer a common evolutionary history without consideration of WGS analysis.

**Fig. 2.**
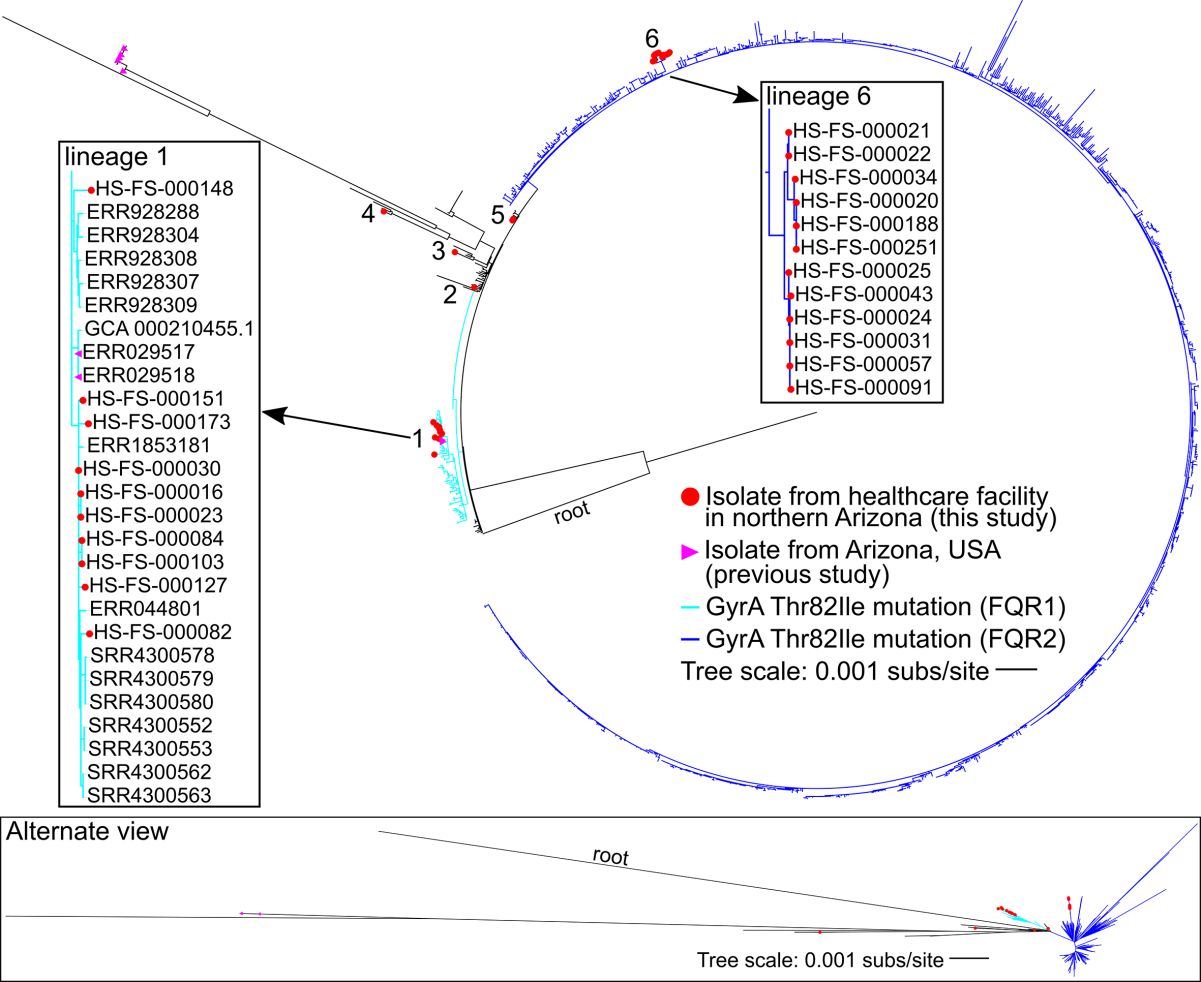
Maximum-likelihood phylogeny of *C. difficile* ST1 genomes (*n*=1379). Clinical isolates from healthcare facilities in northern Arizona (this study, in red) are present in six different lineages (1–6). The presence of the GyrA Thr82Ile mutation conferring quinolone resistance is indicated by blue branches (light blue indicates FQR1 and dark blue indicates FQR2 identified in a previous study [9]). Seven isolates from Arizona sequenced as part of previous studies are identified with purple triangles. Boxes highlight lineages (labelled 1 and 6) that contain multiple isolates from healthcare facilities in northern Arizona.

An additional phylogenetic analysis was conducted for only ST1 genomes (*n*=1379). A maximum-likelihood phylogeny (Fig. 2) was inferred on a concatenation of 4283 SNPs (Table S3). The consistency index (excluding parsimony-uninformative SNPs) was 0.87 and the retention index was 0.98. The GyrA Thr82Ile mutation conferring fluoroquinolone resistance is conserved in two lineages labelled FQR1 and FQR2 [9]. Isolates from two northern Arizona hospitals (*n*=27) that were sequenced as part of this study are found in FQR1 and FQR2 as well as lineages that do not have the GyrA Thr82Ile mutation. The northern Arizona isolates group into six independent lineages (labelled 1–6 in Fig. 2), separated by isolates collected from diverse geographical locations demonstrating that at least six separate introductions of ST1 isolates have occurred in the healthcare facilities over an 18-month time frame (Table 1, Fig. 2). Lineages that included more than one northern Arizona isolate [1, 5, 6] were investigated further to determine the number of SNPs differentiating strains within each lineage (pairwise comparisons of high-quality positions in non-repetitive regions against CD196 reference). Northern Arizona isolates within lineage 1, which also included isolates from other regions, vary by 0–31 SNPs (called from 3 617 365 core genomic positions). Isolates from Arizona sequenced as part of previous studies (*n*=7) [9, 83] are present in two lineages of the ST1 phylogeny, including lineage 1. These previously sequenced isolates from Arizona are from human and food sources [9, 84], and pairwise comparisons indicate that the Arizonan isolates from previous studies within lineage 1 of Fig. 2 (collected from food samples in 2007) are separated from northern Arizona isolates (this study) by 8–22 SNPs (called from 3 617 365 core genomic positions). Lineage 5 includes two northern Arizona isolates collected from the same patient; the isolates had no core genomic SNP differences. Lineage 6 is a monophyletic lineage that includes only northern Arizona isolates (this study); the genomes within this lineage vary by 0–17 pairwise SNPs (called from 3 895 562 core genomic positions). The average inter-lineage (lineages 1–6) distance between northern Arizona isolates sampled in this study is 136 SNPs.

### Timing

Bayesian analysis of SNP data (with recombination removed) representing a set of ST1 and outgroup genomes (*n*=90) for which sample dates are known estimated the SNP accumulation rate at 1.13E-7 substitutions per site per year [highest posterior density (HPD) interval: 8.0632E-8–1.4634E-7]. Considering the size of the *C. difficile* genome, this rate correlates to less than one SNP per genome per year. This SNP accumulation rate is slightly lower than a previous estimate for ST1 [9], as well as within-host rates for *C. difficile* isolates from clinical samples [26, 85]. The estimated divergence time for the ST1 lineage genomes included in our analysis is 40.06 years ago (95 % HPD interval between 32.14 and 49.54 years).

### Analysis of *C. difficile* transmission and persistence

**Fig. 3.**
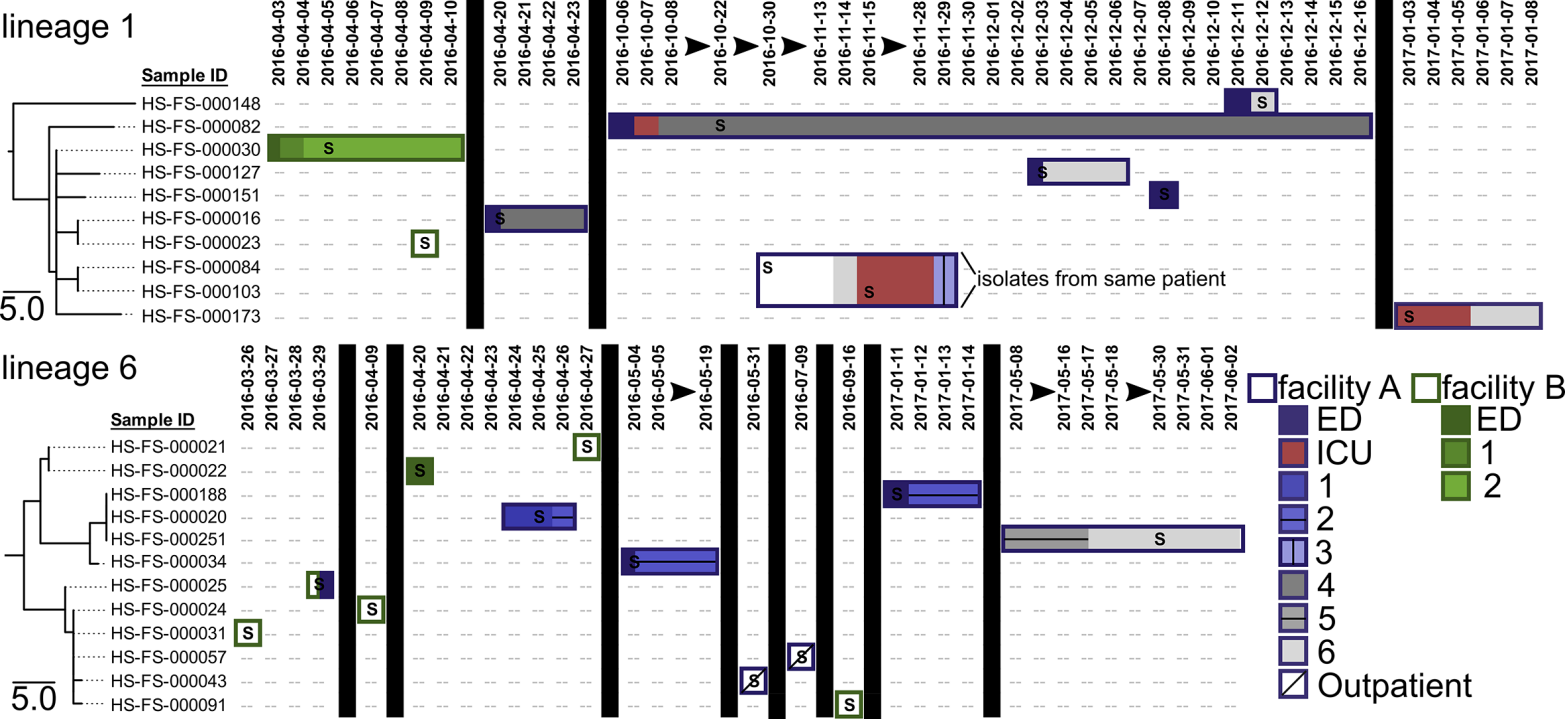
Maximum-parsimony phylogenies for northern Arizona isolates within lineages 1 and 6 identified in Fig. 2 paired with patient location information to assess potential *C. difficile* ST1 transmission and persistence within and among facilities. *C. difficile* ST1 isolates originated from patient faecal samples collected at two facilities in the same healthcare network (facility A in purple, facility B in green). The location of the patient from which isolates originated is mapped over time (date formatting: YYYY-MM-DD) to the right of the phylogeny using rectangles. Patient locations at facility A are indicated with purple outlined rectangles whereas patient locations at facility B are indicated by rectangles outlined in green. Different wards within the facilities are indicated with differential shadings and patterns. Vertical black boxes and black arrows illustrate breaks in time. The letter ‘S’ indicates the date of collection for the faecal sample from which each isolate originated. Isolates HS-FS-000084 and HS-FS-000103 originated from the same patient. The patient from which isolate HS-FS-000025 originated was transferred between facilities. The analysis identifies potential transmission of *C. difficile* within the healthcare network and persistence of a genotype within a patient.

Core genome SNP phylogenies for isolates collected from healthcare facilities in this study were paired with patient epidemiological data to provide insight into *C. difficile* prevalence, acquisition and transmission within and between the two facilities (labelled A and B) (Fig. 3). The estimated evolutionary rate within *C. difficile* ST1 genomes suggests that very few SNPs separate isolates involved in recent transmission events, which has been observed previously [26]. Patient location information reveals that patients with CDI-associated diarrhoea were frequently moved to multiple locations within a facility and once between facilities. Northern Arizona isolates within lineage 1 are closely related, and isolates within lineage 6 are also differentiated by very few SNPs (Fig. 2). Isolates separated by zero SNPs are observed at the same healthcare facility at multiple timepoints (e.g. lineage 6: HS-FS-000188, HS-FS-000020, HS-FS-000251 in Fig. 3) and across both healthcare facilities (e.g. lineage 1: HS-FS-000016, HS-FS-000023; lineage 6: HS-FS-000024, HS-FS-000031, HS-FS-000057 in Fig. 3). Isolates HS-FS-000057 and HS-FS-000043 are separated by 1 SNP and the two patients from which these isolates were sampled resided at the same skilled nursing facility, which is a third and separate entity from the two facilities at which samples were collected in this study. Thus, our analysis identifies potential transmission of *C. difficile* within the healthcare network. Some isolates (e.g. HS-FS-000148 in lineage 1) are more distantly related to other sampled isolates, which could indicate infections acquired from community or environmental reservoirs. *C. difficile* within lineages 1 and 6 appear to be prevalent in northern Arizona (although sampling in this study is limited to clinical specimens from two facilities) and are perhaps circulating within healthcare facilities.

On two occasions, multiple isolates were obtained from the same patient. Isolates HS-FS-00084 and HS-FS-000103 (lineage 1, Fig. 2) were collected from the same patient 16 days apart; isolates HS-FS-000264 and HS-FS-000287 (lineage 5, Fig. 1) were collected from a different patient 1 month apart. In both cases, the isolates sampled from the same patient were separated by zero SNPs, indicating persistent infections or re-infections from the same source rather than new infections with a new genotype.

### *In silico* predicted AMR profiles in ST1 genomes

To understand AMR among northern Arizona isolates as well as ST1 in general, all ST1 genomes were screened against 2177 proteins in the CARD database with LS-BSR (Table S8). The results indicate that although no proteins had hits (BSR value >0.9) across all ST1 genomes, several proteins were highly conserved across many ST1 isolates. The efflux transporter encoded by the *C. difficile cdeA* gene is highly conserved across ST1 genomes (average BSR value 0.99, BSR values >0.9 in 1375 of 1379 ST1 genomes); this CDS is also present in all northern Arizona isolates (Table 1). Although over-expression of the protein in *Escherichia coli* has been correlated with increased fluoroquinolone (norfloxacin and ciprofloxacin) resistance, the role of the CdeA protein in *C. difficile* is unclear [86]. Some isolates such as CD196 have high BSR values for the CdeA protein but are susceptible to some fluoroquinolones (gatifloxacin, moxifloxacin, levofloxacin [2]), indicating the presence of this gene alone does not infer resistance to newer fluoroquinolones in ST1 genomes. Genes encoding proteins associated with TetM and ErmB genetic elements previously described to confer resistance to tetracycline and erythromycin in *C. difficile* [36, 87–89] are present in a number of ST1 genomes but are not universally conserved (TetM present in ~7 % of ST1 genomes, ErmB present in ~8 %). The *tetM* gene is present in one of the 27 northern Arizona isolates whereas the *ermB* gene is present in 10 northern Arizona isolates (all identified as FQR1) (Table 1). A gene encoding a dihydrofolate reductase (DfrF) protein was identified in 10 ST1 genomes including one isolate from northern Arizona (Table 1). DfrF has been associated with resistance to trimethoprim in *Enterococcus faecalis* [90] and *Streptococcus pyogenes* [91]. Proteins encoded by the *vanG* operon associated with vancomycin resistance in *E. faecalis* (MIC 16 µg ml^−1^) [92] were identified for two ST1 genomes (BSR values ranging from 0.92 to 1), including one isolate from northern Arizona (HS-FS-000151). These genes were identified in addition to the vanG-like gene cluster identified in *C. difficile* [89], which is highly conserved among *C. difficile* genomes.

Mobile genetic elements are common within *C. difficile* genomes and have been associated with AMR [72, 73]; therefore, AMR markers identified by LS-BSR were evaluated in the context of transposons. The *ermB* gene present in 10 isolates from this study was associated with Tn6194 (HG475346.1) that has been demonstrated to be transferred between *C. difficile* and *E. faecalis* [93] (Table S9). The *tetM* gene present in one northern Arizona isolate is not clearly associated with any of the previously described transposons. The contig on which the *tetM* gene is located shares high identity with some *Enterococcus faecium* genomes (e.g. accession CP020488, locus tags B6S06_08495 – B6S06_08555) and with a genomic region of RT017 *C. difficile* strain DSM 29627 (CP016102, CDIF29627_00594 – CDIF29627_00604) [94]. This region is also present in a number of ST1 genomes (publicly available SRA data included in this study) containing the *tetM* gene that do not show strong evidence of containing any of the screened AMR-associated transposons; this suggests the presence of a new and uncharacterized transposon in *C. difficile* ST1.

In addition to gene presence/absence, several SNP mutations have been described that confer resistance to quinolones [36, 40, 41, 74]. A comparison of SNP calls across all *C. difficile* genomes at those positions demonstrated that >95 % of ST1 genomes contained the GyrA Thr82Ile mutation that confers quinolone resistance, whereas only ~12 % of non-ST1 *C. difficile* genomes screened in this study have this mutation. Interestingly, genomic data for five of the 27 isolates from CDI cases at northern Arizona facilities indicate these isolates do not have the GyrA Thr82Ile mutation conferring quinolone resistance (lineages 2–5 in Fig. 2). Additional mutations in GyrA and GyrB associated with quinolone resistance were not broadly conserved in ST1 genomes (Table S10). These results suggest that AMR varies among ST1 isolates, including among northern Arizona isolates from the same hospital.

### AMR testing

Four northern Arizona isolates (this study) with varied *in silico* predicted AMR profiles (Table 1) were screened for AMR (ciprofloxacin, tetracycline, vancomycin) using Etests (Table S11). These four isolates (HS-FS-000082, HS-FS-000127, HS-FS-000151, HS-FS-000264) were chosen to represent the diversity of *in silico* predicted AMR profiles among northern Arizona isolates (Table 1). As with most *C. difficile* isolates [36], all four isolates were resistant to ciprofloxacin (MIC >32 µg ml^−1^), which is a commonly prescribed fluoroquinolone. One isolate (HS-FS-000082) was also resistant to tetracycline (MIC >32 µg ml^−1^) and this isolate contained the *tetM* gene (described above). Three of the four isolates had intermediate resistance (MIC 4 µg ml^−1^) to vancomycin as has been observed in other *C. difficile* isolates [95, 96]. One of these isolates (HS-FS-000151) with intermediate vancomycin resistance contained CDSs associated with a *vanG* operon in *E. faecalis* (described above); however, this region did not appear to confer increased resistance to vancomycin in the tested isolate.

### Pan-genome composition of ST1 genomes

LS-BSR analyses were performed to understand the size and extent of the ST1 core and pan-genome, to identify CDSs that may contribute to the association of ST1 with the hospital environment, and to evaluate potentially unique features of northern Arizona isolates. The strict core genome of 1379 *C* *. difficile* ST1 genome assemblies was determined by LS-BSR to be 1991 CDSs (Table S12), which corresponds to approximately 55 % of the total CDSs in the genome for ST1 strain CD196. The analysed ST1 genomes spanned a wide range of assembly quality, which could result in underestimation of the core genome size. However, evaluating CDSs that are highly conserved among ST1 genomes could provide insight into why the lineage is so successful in addition to fluoroquinolone resistance. BSR values were used to identify CDSs that have been gained or lost in ST1 genomes. CDSs (*n*=44) were identified as highly conserved among ST1 genomes and largely absent from non-ST1 genomes, whereas 14 CDSs were identified as being lost from ST1 genomes (highly conserved among non-ST1 genomes and largely absent from ST1 genomes, see Methods for criteria) (Table S13). CDSs identified as highly conserved in ST1 include some genomic regions previously identified as unique to RT027 isolates in a comparison of three genomes [2]. For example, CDSs associated with the insertion of a catalytically more efficient *thyA* gene that disrupts the *thyX* gene in previously studied RT027 isolates [97] are highly conserved in the ST1 genomes analysed here; these CDSs were upregulated by an ST1/RT027 isolate during the early stage of an infection in a monoxenic mouse model [98]. Additional CDSs highly conserved in ST1 genomes are annotated as transposases or have unknown functions (Table S13). Binary toxin genes (*cdtA* and *cdtB*) are highly conserved within ST1 and largely absent from many other STs but did not meet the threshold defined here to be included as gained/lost CDSs.

To understand if the phenomenon of gene acquisition/loss is common across all lineages of *C. difficile *, a comparable analysis was conducted on ST8 (RT002 and RT159) genomes (*n*=31). The results demonstrate that although no CDSs are unique to ST8, 30 CDSs are differentially conserved in ST8 and two CDSs are generally deleted (see Methods for criteria). Genomes (*n*=13) from ST63 (RT053), which have been shown to contain similar *in silico* predicted AMR profiles (GyrA mutation) to ST1 (see below), were also compared to understand CDS conservation and loss. The results demonstrate that 26 CDSs are highly conserved among ST63 genomes and no regions appear to be specifically lost by this lineage. For genomes (*n*=34) identified as ST15 (RT010), which includes non-toxigenic strains, 118 CDSs were identified as highly conserved and 26 CDSs were identified as generally deleted. These analyses may suggest that ST15 and ST1 are adapting to different niches/environments. ST1 may be adapting to the human gut, and a large percentage of isolates from patients with CDI in northern Arizona belong to ST1.

Although ST11 (RT078) genomes were excluded from the initial round of this analysis due to filtering genome assemblies by mash distance, CDSs identified as highly conserved or generally lost within ST1 were compared to ST11 lineage genomes (*n*=15, accession numbers in Table S13). The ST11 lineage has been associated with severe disease and has been commonly isolated from clinical samples as well as agricultural, animal and retail meat samples [84, 99–104]. Several CDSs identified as highly conserved within ST1 were also conserved in ST11 genomes (Table S13). These CDSs include a DNA-binding regulator, a histidine kinase and an RNA polymerase sigma factor. Some CDSs identified as generally lost within ST1 are conserved within ST11 (e.g. a TetR/AcrR family transcriptional regulator).

Although LS-BSR analysis indicates that over half of the predicted CDSs present in the genome for strain CD196 are part of the strict core genome for ST1 isolates, some of the CDSs present in this genome (and others within ST1) are differentially conserved throughout the ST1 lineage. Some CDSs are lineage-specific or have been lost in particular lineages of ST1 (Fig. S1). In total, 5937 CDSs were identified in the accessory genome for ST1 and 1049 CDSs were identified as unique to one genome. This variation in genomic content within the ST1 lineage suggests that different lineages/isolates within ST1 will vary phenotypically, which could impact AMR, transmission and virulence. For example, Stone and colleagues [105] found variable toxin production between two ST1 isolates using a transepithelial electrical resistance (TEER) assay. To understand the genetic composition of northern Arizona isolates, LS-BSR was used to identify any unique CDSs within lineages 1–6 in Fig. 2 compared to other ST1 isolates, but no CDSs were found to be unique to any of these lineages. However, isolates HS-FS-000082 and HS-FS-000151, both within lineage 1, each contained a CDS not identified in any other ST1 genome (HS-FS-000082 – ABC transporter permease, HS-FS-000151 – site-specific integrase putatively associated with a second copy of *ermB* in this genome).

Two northern Arizona isolates (HS-FS-000084, HS-FS-000103; lineage 1 in Fig. 2) that originated from the same patient over a 16-day period had smaller assembly sizes than many other ST1 genomes. These two genome assemblies are ~160 kb smaller than the average size of ST1 genomes included in this study and are ~210 kb smaller than other genomes within lineage 1 of Fig. 2. LS-BSR analysis indicated that 65 CDS are highly conserved in all other ST1 genomes but are absent in these two genome assemblies (Table S14). Poor assembly quality could result in a smaller genome with fewer CDSs; however, read mapping against the complete genome for CD196 identified a chromosomal region (~155 kb) that is present in all other ST1 genomes but is absent in the two isolates collected from the same patient. The 65 CDSs absent from the two genomes share high identity to CDSs within this chromosomal region of CD196 (Table S14). Interestingly, many of these CDSs are associated with carbohydrate metabolism and the phosphotransferase system (PTS). The PTS can impact regulation of many cellular processes [106] and differential expression of PTS genes by *C. difficile* has been associated with nutrition shifts *in vitro* [107, 108] and during the course of mouse infection studies [98].

A set of 52 peptide sequences [2] previously associated with differential conservation in ST1 genomes (Table S6) were screened against all genomes with LS-BSR. The results indicate that several features associated with an epidemic ST1 strain are also present in non-ST1 genomes (Table S15) or are not highly conserved among other ST1 genomes. Although this result does not rule out that these regions are associated with virulence in some ST1 strains, it does suggest that these regions do not fully explain the success of the ST1 lineage. Collins and colleagues [18] identified genomic features associated with increased metabolism of trehalose in epidemic ribotypes. A mutation in the TreR protein (Leu172Ile) associated with trehalose metabolism is present in predicted proteins for 1375 of 1379 screened ST1 genome assemblies. Truncated predicted proteins were identified for two ST1 genome assemblies (ERR030337, GCA_900011385.1), and predicted proteins with low identity to the TreR protein for *C. difficile* strain CD196 (CBA65726.1) were identified for two genome assemblies isolated from northern Arizona (HS-FS-000084, HS-FS-000103; isolates from the same patient – see above and lineage 1 in Fig. 2). Four protein sequences associated with trehalose metabolism in RT078 (ST11) isolates [18] were also screened against genome assemblies. High BSR values (≥0.96) for all four protein sequences were identified for genome assemblies representing multiple MLST sequence types (Table S15), including three ST1 genome assemblies (ERR026353, ERR030384, ERR251831; in-house assemblies from publicly available sequencing read data). The four protein sequences were not identified for any of the 27 sequenced northern Arizona isolates.

### *In silico* ribotyping

The gold standard for *C. difficile* genotyping has been ribotyping, wherein RT027 isolates are primarily associated with ST1 [22]. Here we have focused on ST1, which forms a monophyletic clade within a whole genome SNP phylogeny (Fig. 1). However, there appears to be a disconnect in the literature between researchers using ribotyping and those using either MLST or WGS approaches. To relate northern Arizona isolates to published *C. difficile* PCR ribotypes without the need for performing PCR ribotyping in the laboratory, we explored the potential to extract ribotyping profiles from sequence data. To examine the utility of an *in silico* ribotyping approach, raw sequencing reads representing 4643 genomes (initial test set) from the SRA were aligned against amplicons predicted by probing standard ribotyping primers against three finished ST1 genomes, and hits were identified from samples with even read mapping across all amplicons (see Methods). An analysis of different thresholds for variable read mapping demonstrated that >20 % of the maximum read counts was an appropriate threshold for calling ribotype amplicons (Table S16). Of the 4643 genomes initially screened, 1241 were sequence typed as ST1 using stringMLST, and 1226 of these ST1 genomes were identified as RT027 based on *in silico* ribotype profiles (Table S17). The *in silico* ribotyping method correctly identified ~99 % of ST1 genomes based on their ribotype profiles. Only 17 of 3402 non-ST1 genomes were identified as ST1. Importantly, the PCR ribotypes for genomes in this data set were not evaluated, and it is assumed here that all ST1 genomes analysed are RT027 and all non-ST1 genomes are not RT027. ST1 also includes RT016, 036 and 176, but predominantly RT027 isolates have been associated with ST1.

To further evaluate the utility of the *in silico* ribotyping approach, the methodology was applied to a set of sequencing reads representing 624 isolates that were PCR ribotyped in a recently published study [81]. Of the 624 isolates, 216 were PCR ribotyped as RT027 and all of these isolates were *in silico* ribotyped as RT027. Additionally, 21 PCR RT176 isolates, which are associated with the ST1 lineage, were included in this data set. Of these 21 isolates, only one was *in silico* ribotyped as RT027 (Table S17). No other isolate was incorrectly classified as RT027 *in silico*. The *in silico* ribotyping approach was then applied to isolates sequenced as part of this study, and all 27 northern Arizona isolates were *in silico* ribotyped as RT027 (Table S17). All northern Arizona isolates were also sequence typed as ST1 (*in silico*) according to the MLST scheme for *C. difficile *.

PCR ribotyping was performed on 10 *C* *. difficile* isolates with various sequence types for comparison with *in silico* results (Fig. S2). Two ST1 isolates presented similar PCR ribotyping profiles, and PCR ribotyping profiles for these two ST1 isolates were comparable to *in silico* ribotyping results, although not exact matches – this is partly due to primer selection for *in silico* and PCR ribotyping methods. The *in silico* profile included seven bands; one ST1 isolate presented analogues to all seven bands plus one larger band whereas the other ST1 isolate presented six analogous bands plus one larger band (Table S18). Variation between ribotyping profiles predicted from four RT027 genome assemblies and actual ribotyping profiles has been reported previously [109]. Non-ST1 isolates produced dissimilar PCR ribotyping profiles to ST1 isolates, and very few non-ST1 isolates were identified as ST1 by the *in silico* ribotyping method. The *in silico* ribotyping method described here could potentially provide a means to connect ribotyping information from past studies to the expanding WGS data available for *C. difficile* isolates.

## Discussion

*C. difficile* ST1 is a successful worldwide lineage that appears to be especially adapted to the human gut and healthcare environments. The rise of *C. difficile* in hospitals globally has been attributed, at least partially, to the emergence and proliferation of ST1 (RT027) [110]. In this study, we sequenced 27 ST1 isolates collected between March 2016 and September 2017 from two healthcare facilities in northern Arizona (these 27 isolates represent ~15 % of all isolates collected as part of a larger surveillance study) and compared them to a worldwide collection of *C. difficile* ST1 genomes. The results demonstrate that diverse ST1 isolates were present in the sampled healthcare facilities; northern Arizona isolates are distributed throughout the ST1 phylogeny, including within two previously identified fluoroquinolone resistant lineages (FQR1 and FQR2 [9]*)*. At least six separate introductions (lineages) of *C. difficile* ST1 into the two sampled healthcare facilities were observed over the period of surveillance (Fig. 2). Four of the introductions/lineages are represented by only one isolate or genotype (lineages 2–5 in Fig. 2), whereas lineages 1 and 6 contain multiple northern Arizona genotypes (defined as SNP variation among isolates). Lineage 6 is a distinct lineage that includes only isolates from northern Arizona facilities. Interestingly, two isolates collected from food samples in Arizona as part of previous studies [9, 84] are closely related to some of the clinical isolates sequenced in this study (lineage 1 in Fig. 2). *C. difficile* within lineages 1 and 6 appear to be prevalent ST1 genotypes circulating within northern Arizona.

WGS provides a high-resolution method for differentiating bacterial isolates and offers insight into how pathogens persist and are transmitted within healthcare networks. Several studies, including this one, have indicated that the SNP accumulation rate in *C. difficile* is approximately one SNP per genome per year [26, 85]; however, SNP accumulation rates can be complicated for bacteria with a spore stage in their life cycle [111]. Bayesian analysis suggests that the divergence time for the ST1 lineage is approximately 40 years ago (95 % HPD interval between 32.14 and 49.54 years), which is consistent with the identification of the first ST1/RT027 isolate in 1985 [2, 3]. He and colleagues [9] analysed a data set of ST1 genomes and described two epidemic lineages, both of which acquired the GyrA Thr82Ile mutation conferring quinolone resistance; the time of emergence for these two ST1 lineages was estimated to be in the early 1990s. The SNP accumulation rate within *C. difficile* must be considered when attempting to track isolates on an epidemiological time scale [112] and cases linked by recent transmission will probably be separated by very few SNPs [26]. The genomic variation among northern Arizona isolates suggests that although some CDI cases from which these isolates were collected may potentially be the result of transmission within healthcare facilities, other cases are seemingly unrelated. We observed that closely related isolates, based on WGS analysis, were sampled in patients at multiple facilities and over multiple time points. In two instances, WGS showed that the same genotype persisted in a patient rather than the patient being infected with a different genotype. Since patients with CDI are moved between facilities and similar *C. difficile* genotypes are detected at different facilities, infection prevention strategies must be implemented across the entire healthcare network to be most effective. Putatively unrelated cases may be the result of particular ST1 genotypes circulating in northern Arizona reservoirs and emerging as CDI cases due to antibiotic use or other factors. Importantly, monitoring transmission and persistence of these ST1 isolates with sub-genomic methods, such as ribotyping or MLST, would not have been possible due to a lack of resolution provided by these methods. Incorporating WGS and comparative genomics into the healthcare network surveillance programme enabled more effective monitoring, which can hopefully improve patient health in the future.

AMR varies within *C. difficile* [36], and *in silico* predicted AMR profiles for ST1 isolates sampled in this study are also variable. Resistance to fluoroquinolones within *C. difficile* ST1 has been the focus of many studies [4, 9, 37–39] and approximately 95 % of the ST1 genomes compared in this study have the missense mutation in the *gyrA* gene (Fig. 2, Table S10) linked to resistance to some fluoroquinolones. The use of quinolones to treat unrelated infections may select for ST1, supporting the proliferation of clinical CDI caused by this sequence type. Interestingly, the GyrA Thr82Ile mutation and other mutations known to confer quinolone resistance were absent in five of the 27 ST1 isolates collected in this study. These five isolates account for four introductions of the ST1 lineage into the healthcare network in this region (lineages 2–5 in Fig. 2); one facility within the healthcare network implemented a programme to restrict fluoroquinolone use in 2017, which may reduce selection for the GyrA Thr82Ile genotype. AMR screening of northern Arizona isolates with varying *in silico* predicted AMR profiles (Table S11) indicated that all isolates were resistant to ciprofloxacin; resistance to ciprofloxacin is common among *C. difficile* [36]. Resistance to tetracycline was indicated for one northern Arizona isolate for which a *tetM* gene was identified in the genome. Use of tetracyclines has not been frequently linked to CDI [113]; however, tetracycline resistance has been associated with some lineages of *C. difficile* such as RT078 (ST11) [114–116]. No strains were fully resistant to vancomycin, which is a recommended treatment option for CDI [42], despite the presence of CDSs associated with vancomycin resistance being detected in one tested genome. Continued screening of *C. difficile* AMR is important to monitor AMR variability and provide insight for updating treatment guidelines.

Although AMR plays an important role in *C. difficile* epidemiology, other factors, in addition to quinolone resistance, probably contribute to the prevalence of CDI attributed to ST1. For example, the GyrA Thr82Ile mutation is also highly conserved in ST63 (RT053) genomes, although this ST is not as successful as ST1 based on reported genotype frequencies from hospital isolation [11, 13, 26, 27]. Additionally, *C. difficile* ST1 appears to be less prevalent than other STs in many sampled environments (samples not associated with the human gut or hospital) based upon environmental survey studies [30, 31, 117]. LS-BSR analysis indicated that genomic regions were highly conserved within ST1 genomes, as well as a number of regions that have been broadly deleted within ST1 genomes (Table S13). Reduction of the core genome can be associated with niche differentiation [118, 119]. Interestingly, two isolates from the same patient (this study) demonstrated large deletions compared to other ST1 genomes. The observed genome region acquisition and loss across ST1 may be associated with its prevalence in the human/hospital environment. An analysis of other STs demonstrates that the gene gain/loss may be more common in ST1 than other toxigenic lineages. However, as most sampling efforts are focused on clinical settings, the prevalence of ST1 in other environments is not fully understood. Genetic variability within ST1 may also contribute to the success of the lineage. For instance, resistance to clindamycin is not universal within ST1 [4, 120] but would provide an advantage to some ST1 isolates. Genomics alone cannot definitively identify why ST1 is such a successful lineage of *C. difficile *, but can identify targets for further investigation (e.g. CDSs identified in Table S13). Additionally, here we focus on bacterial genomics without consideration of factors such as variation in gene regulation, host immune response, or hospital practices such as antimicrobial use policies.

*C. difficile* isolates have been differentiated using a variety of methods including MLST and PCR ribotyping. In this study, we used the MLST designation, as we can easily genotype sequenced genomes *in silico* in the absence of ribotyping data. However, due to recombination, phylogenies inferred from a concatenation of MLST markers have been shown to be incongruent with WGS phylogenies [121]. Using WGS data for *C. difficile *, we demonstrate that multiple STs are paraphyletic on the core genome SNP phylogeny (Fig. 1). This is important if common STs are expected to have a shared evolutionary history, yet the history is conflicting when using the entire genome. Ribotyping represents a legacy method that is still used to type isolates and infer relationships. However, there remains a disconnect between ribotyping and sequencing methods. In this study, we present a workflow that can associate raw sequence data to a ribotype profile (RT027). The amplicons that were used for *in silico* ribotyping were identified from only three complete ST1 genomes; draft genomes are not useful for the identification of predicted amplicons due to the potential collapse of repeat regions (e.g. 16S rRNA genes) [122]. As additional complete genomes are generated from diverse ribotypes, this method can be modified to associate ribotype information with whole genome sequence and MLST data, which is useful for bridging between different methodologies. WGS and analysis provides the highest resolution method for comparing *C. difficile* isolates and should be included in epidemiological studies if possible.

This study has provided a large-scale analysis of a single sequence type of *C. difficile* in order to understand the diversity within a successful, world-wide lineage and place isolates from a local healthcare network into a global context. The analyses indicated multiple introductions of *C. difficile* ST1 into the healthcare network, provided insight into potential transmission of *C. difficile* within the healthcare network and identified AMR variation among isolates. The genomic diversity within the ST1 genomes suggests that broadly applying labels to ST1 genomes, such as hypervirulent clone, may be inappropriate without an in-depth analysis of the gene presence/absence and SNP markers for AMR. Additional studies into how genomic variation affects toxin production and virulence are needed to assess the phenotypic diversity of ST1 as it relates to the observed genotypic diversity; these studies are currently ongoing and will help shape how we approach studies using sub-genomic information, such as ribotyping and MLST.

## Data Bibliography

1. A full listing of NCBI accessions for strains used in this paper is available in Table S1.
  2. Previously identified genomic regions associated with C. difficile ST1 virulence used in analyses in this study are provided in Table S6 or in the manuscript text.
  3. An in silico polymerase chain reaction script used in this study is available at GitHub – https://github.com/TGenNorth/vipr https://github.com/TGenNorth/vipr.
  4. An in silico MLST script used in this study is available at GitHub – (https://gist.github.com/jasonsahl/2eedc0ea93f90097890879e56b0c3fa3).

## Supplementary Data

Supplementary File 1

Supplementary File 2

Supplementary File 3

Supplementary File 4

Supplementary File 5

Supplementary File 6

Supplementary File 7

Supplementary File 8

Supplementary File 9

Supplementary File 10

Supplementary File 11

Supplementary File 12

Supplementary File 13

Supplementary File 14

Supplementary File 15

Supplementary File 16

Supplementary File 17

Supplementary File 18

Supplementary File 19

## References

[1] Lawson PA, Citron DM, Tyrrell KL, Finegold SM (2016). Reclassification of *Clostridium difficile* as *Clostridioides difficile* (Hall and O'Toole 1935) Prévot 1938. Anaerobe.

[2] Stabler RA, He M, Dawson L, Martin M, Valiente E (2009). Comparative genome and phenotypic analysis of *Clostridium difficile* 027 strains provides insight into the evolution of a hypervirulent bacterium. Genome Biol.

[3] Popoff MR, Rubin EJ, Gill DM, Boquet P (1988). Actin-specific ADP-ribosyltransferase produced by a *Clostridium difficile* strain. Infect Immun.

[4] McDonald LC, Killgore GE, Thompson A, Owens RC, Kazakova SV (2005). An epidemic, toxin gene-variant strain of *Clostridium difficile*. N Engl J Med.

[5] Loo VG, Poirier L, Miller MA, Oughton M, Libman MD (2005). A predominantly clonal multi-institutional outbreak of *Clostridium difficile*-associated diarrhea with high morbidity and mortality. N Engl J Med.

[6] Warny M, Pepin J, Fang A, Killgore G, Thompson A (2005). Toxin production by an emerging strain of *Clostridium difficile* associated with outbreaks of severe disease in North America and Europe. The Lancet.

[7] Barbut F, Mastrantonio P, Delmée M, Brazier J, Kuijper E (2007). Prospective study of *Clostridium difficile* infections in Europe with phenotypic and genotypic characterisation of the isolates. Clin Microbiol Infect.

[8] Kuijper EJ (2008). Update of *Clostridium difficile* infection due to PCR ribotype 027 in Europe, 2008. Euro Surveill.

[9] He M, Miyajima F, Roberts P, Ellison L, Pickard DJ (2013). Emergence and global spread of epidemic healthcare-associated *Clostridium difficile*. Nat Genet.

[10] Wilcox MH, Shetty N, Fawley WN, Shemko M, Coen P (2012). Changing epidemiology of *Clostridium difficile* infection following the introduction of a national ribotyping-based surveillance scheme in England. Clin Infect Dis.

[11] Tickler IA, Goering RV, Whitmore JD, Lynn ANW, Persing DH (2014). Strain types and antimicrobial resistance patterns of *Clostridium difficile* isolates from the United States, 2011 to 2013. Antimicrob Agents Chemother.

[12] Davies KA, Ashwin H, Longshaw CM, Burns DA, Davis GL (2016). Diversity of *Clostridium difficile* PCR ribotypes in Europe: results from the European, multicentre, prospective, biannual, point-prevalence study of *Clostridium difficile* infection in hospitalised patients with diarrhoea (EUCLID), 2012 and 2013. Euro Surveill..

[13] Rupnik M, Tambic Andrasevic A, Trajkovska Dokic E, Matas I, Jovanovic M (2016). Distribution of *Clostridium difficile* PCR ribotypes and high proportion of 027 and 176 in some hospitals in four South Eastern European countries. Anaerobe.

[14] Giancola SE, Williams RJ, Gentry CA (2018). Prevalence of the *Clostridium difficile* BI/NAP1/027 strain across the United States Veterans Health administration. Clin Microbiol Infect.

[15] Aptekorz M, Szczegielniak A, Wiechuła B, Harmanus C, Kuijper E (2017). Occurrence of *Clostridium difficile* ribotype 027 in hospitals of Silesia, Poland. Anaerobe.

[16] Karlowsky JA, Adam HJ, Kosowan T, Baxter MR, Nichol KA (2018). PCR ribotyping and antimicrobial susceptibility testing of isolates of *Clostridium difficile* cultured from toxin-positive diarrheal stools of patients receiving medical care in Canadian hospitals: the Canadian Clostridium difficile surveillance study (CAN-DIFF) 2013-2015. Diagn Microbiol Infect Dis.

[17] Yakob L, Riley TV, Paterson DL, Marquess J, Magalhaes RJS (2015). Mechanisms of hypervirulent *Clostridium difficile* ribotype 027 displacement of endemic strains: an epidemiological model. Sci Rep.

[18] Collins J, Robinson C, Danhof H, Knetsch CW, van Leeuwen HC (2018). Dietary trehalose enhances virulence of epidemic *Clostridium difficile*. Nature.

[19] Bidet P, Barbut F, Lalande V, Burghoffer B, Petit JC (1999). Development of a new PCR-ribotyping method for *Clostridium difficile* based on ribosomal RNA gene sequencing. FEMS Microbiol Lett.

[20] Martin JSH, Monaghan TM, Wilcox MH (2016). *Clostridium difficile* infection: epidemiology, diagnosis and understanding transmission. Nat Rev Gastroenterol Hepatol.

[21] Knetsch CW, Terveer EM, Lauber C, Gorbalenya AE, Harmanus C (2012). Comparative analysis of an expanded *Clostridium difficile* reference strain collection reveals genetic diversity and evolution through six lineages. Infection, Genetics and Evolution.

[22] Griffiths D, Fawley W, Kachrimanidou M, Bowden R, Crook DW (2010). Multilocus sequence typing of *Clostridium difficile*. J Clin Microbiol.

[23] Vanek J, Hill K, Collins J, Berrington A, Perry J (2012). Epidemiological survey of *Clostridium difficile* ribotypes in the North East of England during an 18-month period. J Hosp Infect.

[24] Pituch H, Obuch-Woszczatyński P, Lachowicz D, Wultańska D, Karpiński P (2015). Hospital-based *Clostridium difficile* infection surveillance reveals high proportions of PCR ribotypes 027 and 176 in different areas of Poland, 2011 to 2013. Euro Surveill.

[25] Krutova M, Matejkova J, Nyc O (2014). *C. difficile* ribotype 027 or 176?. Folia Microbiol.

[26] Eyre DW, Cule ML, Wilson DJ, Griffiths D, Vaughan A (2013). Diverse sources of *C. difficile* infection identified on whole-genome sequencing. N Engl J Med.

[27] Dingle KE, Griffiths D, Didelot X, Evans J, Vaughan A (2011). Clinical *Clostridium difficile*: clonality and pathogenicity locus diversity. PLoS One.

[28] Alam MJ, Walk ST, Endres BT, Basseres E, Khaleduzzaman M (2017). Community environmental contamination of toxigenic Clostridium difficile. Open Forum Infect Dis.

[29] Janezic S, Potocnik M, Zidaric V, Rupnik M (2016). Highly divergent *Clostridium difficile* strains isolated from the environment. PLoS One.

[30] Moradigaravand D, Gouliouris T, Ludden C, Reuter S, Jamrozy D (2018). Genomic survey of *Clostridium difficile* reservoirs in the East of England implicates environmental contamination of wastewater treatment plants by clinical lineages. Microb Genom.

[31] Stone NE, Sidak-Loftis LC, Sahl JW, Vazquez AJ, Wiggins KB (2016). More than 50% of *Clostridium difficile* Isolates from Pet Dogs in Flagstaff, USA, Carry Toxigenic Genotypes. PLoS One.

[32] Bartlett JG, Moon N, Chang TW, Taylor N, Onderdonk AB (1978). Role of *Clostridium difficile* in antibiotic-associated pseudomembranous colitis. Gastroenterology.

[33] Larson HE, Price AB, Honour P, Borriello SP (1978). *Clostridium difficile* and the ætiology of pseudomembranous colitis. The Lancet.

[34] George RH, Symonds JM, Dimock F, Brown JD, Arabi Y (1978). Identification of *Clostridium difficile* as a cause of pseudomembranous colitis. Br Med J.

[35] Huang H, Weintraub A, Fang H, Nord CE (2009). Antimicrobial resistance in *Clostridium difficile*. Int J Antimicrob Agents.

[36] Spigaglia P (2016). Recent advances in the understanding of antibiotic resistance in *Clostridium difficile* infection. Therapeutic Advances in Infection.

[37] Emmerson AM, Jones AM (2003). The quinolones: decades of development and use. J Antimicrob Chemother.

[38] Pépin J, Saheb N, Coulombe M-A, Alary M-E, Corriveau M-P (2005). Emergence of fluoroquinolones as the predominant risk factor for *Clostridium difficile*-associated diarrhea: a cohort study during an epidemic in Quebec. Clin Infect Dis.

[39] Linder JA, Huang ES, Steinman MA, Gonzales R, Stafford RS (2005). Fluoroquinolone prescribing in the United States: 1995 to 2002. Am J Med.

[40] Ackermann G, Tang YJ, Kueper R, Heisig P, Rodloff AC (2001). Resistance to moxifloxacin in toxigenic *Clostridium difficile* isolates is associated with mutations in gyrA. Antimicrob Agents Chemother.

[41] Dridi L, Tankovic J, Burghoffer B, Barbut F, Petit J-C (2002). gyrA and gyrB mutations are implicated in cross-resistance to ciprofloxacin and moxifloxacin in *Clostridium difficile*. Antimicrob Agents Chemother.

[42] McDonald LC, Gerding DN, Johnson S, Bakken JS, Carroll KC (2018). Clinical practice guidelines for *Clostridium difficile* infection in adults and children: 2017 update by the infectious diseases Society of America (IDSA) and Society for healthcare epidemiology of America (SheA). Clin Infect Dis.

[43] Smits WK, Lyras D, Lacy DB, Wilcox MH, Kuijper EJ (2016). *Clostridium difficile* infection. Nat Rev Dis Primers.

[44] Gupta A, Jordan IK, Rishishwar L (2017). stringMLST: a fast k-mer based tool for multilocus sequence typing. Bioinformatics.

[45] Bankevich A, Nurk S, Antipov D, Gurevich AA, Dvorkin M (2012). SPAdes: a new genome assembly algorithm and its applications to single-cell sequencing. J Comput Biol.

[46] Ondov BD, Treangen TJ, Melsted P, Mallonee AB, Bergman NH (2016). Mash: fast genome and metagenome distance estimation using MinHash. Genome Biol.

[47] Altschul SF, Madden TL, Schäffer AA, Zhang J, Zhang Z (1997). Gapped blast and PSI-BLAST: a new generation of protein database search programs. Nucleic Acids Res.

[48] Edwards AN, Suárez JM, McBride SM (2013). Culturing and Maintaining *Clostridium difficile* in an Anaerobic Environment. Journal of Visualized Experiments.

[49] Quinlan AR, Hall IM (2010). BEDTools: a flexible suite of utilities for comparing genomic features. Bioinformatics.

[50] Li H (2013). Aligning sequence reads, clone sequences and assembly contigs with BWA-MEM. arXiv.org.

[51] Benson DA, Karsch-Mizrachi I, Clark K, Lipman DJ, Ostell J (2012). GenBank. Nucleic Acids Res.

[52] Sahl JW, Lemmer D, Travis J, Schupp JM, Gillece JD, Roe C, Smith DE, Williamson CHD, Aziz M (2016). NASP: an accurate, rapid method for the identification of SNPs in WGS datasets that supports flexible input and output formats. Microb Genom.

[53] Huang W, Li L, Myers JR, Marth GT (2012). Art: a next-generation sequencing read simulator. Bioinformatics.

[54] Delcher AL, Phillippy A, Carlton J, Salzberg SL (2002). Fast algorithms for large-scale genome alignment and comparison. Nucleic Acids Res.

[55] Nguyen L-T, Schmidt HA, von Haeseler A, Minh BQ (2015). IQ-TREE: a fast and effective stochastic algorithm for estimating maximum-likelihood phylogenies. Mol Biol Evol.

[56] Kalyaanamoorthy S, Minh BQ, Wong TKF, von Haeseler A, Jermiin LS (2017). ModelFinder: fast model selection for accurate phylogenetic estimates. Nat Methods.

[57] Hoang DT, Chernomor O, von Haeseler A, Minh BQ, Vinh LS (2018). UFBoot2: improving the ultrafast bootstrap approximation. Mol Biol Evol.

[58] Schliep KP (2011). phangorn: phylogenetic analysis in R. Bioinformatics.

[59] Farris JS (1989). The retention index and the RESCALED consistency index. Cladistics.

[60] Letunic I, Bork P (2016). Interactive tree of life (iTOL) V3: an online tool for the display and annotation of phylogenetic and other trees. Nucleic Acids Res.

[61] DePristo MA, Banks E, Poplin R, Garimella KV, Maguire JR (2011). A framework for variation discovery and genotyping using next-generation DNA sequencing data. Nat Genet.

[62] McKenna A, Hanna M, Banks E, Sivachenko A, Cibulskis K (2010). The genome analysis toolkit: a MapReduce framework for analyzing next-generation DNA sequencing data. Genome Res.

[63] Bruen TC, Philippe H, Bryant D (2006). A simple and robust statistical test for detecting the presence of recombination. Genetics.

[64] Didelot X, Wilson DJ (2015). ClonalFrameML: efficient inference of recombination in whole bacterial genomes. PLoS Comput Biol.

[65] Rambaut A, Lam TT, Max Carvalho L, Pybus OG (2016). Exploring the temporal structure of heterochronous sequences using TempEst (formerly Path-O-Gen). Virus Evol.

[66] Murray GGR, Wang F, Harrison EM, Paterson GK, Mather AE (2016). The effect of genetic structure on molecular dating and tests for temporal signal. Methods Ecol Evol.

[67] Drummond AJ, Suchard MA, Xie D, Rambaut A (2012). Bayesian phylogenetics with BEAUti and the beast 1.7. Mol Biol Evol.

[68] Baele G, Lemey P, Bedford T, Rambaut A, Suchard MA (2012). Improving the accuracy of demographic and molecular clock model comparison while accommodating phylogenetic uncertainty. Mol Biol Evol.

[69] McArthur AG, Waglechner N, Nizam F, Yan A, Azad MA (2013). The comprehensive antibiotic resistance database. Antimicrob Agents Chemother.

[70] Rasko DA, Myers GSA, Ravel J (2005). Visualization of comparative genomic analyses by blast score ratio. BMC Bioinformatics.

[71] Rasko DA, Rosovitz MJ, Myers GSA, Mongodin EF, Fricke WF (2008). The pangenome structure of Escherichia coli: comparative genomic analysis of E. coli commensal and pathogenic isolates. J Bacteriol.

[72] Brouwer MSM, Warburton PJ, Roberts AP, Mullany P, Allan E (2011). Genetic organisation, mobility and predicted functions of genes on integrated, mobile genetic elements in sequenced strains of Clostridium difficile. PLoS One.

[73] Mullany P, Allan E, Roberts AP (2015). Mobile genetic elements in Clostridium difficile and their role in genome function. Res Microbiol.

[74] Oh H, Edlund C (2003). Mechanism of quinolone resistance in anaerobic bacteria. Clin Microbiol Infect.

[75] Edgar RC (2004). Muscle: a multiple sequence alignment method with reduced time and space complexity. BMC Bioinformatics.

[76] Sahl JW, Caporaso JG, Rasko DA, Keim P (2014). The large-scale blast score ratio (LS-BSR) pipeline: a method to rapidly compare genetic content between bacterial genomes. PeerJ.

[77] Hyatt D, Chen G-L, Locascio PF, Land ML, Larimer FW (2010). Prodigal: prokaryotic gene recognition and translation initiation site identification. BMC Bioinformatics.

[78] Edgar RC (2010). Search and clustering orders of magnitude faster than blast. Bioinformatics.

[79] Kent WJ (2002). BLAT--the BLAST-like alignment tool. Genome Res.

[80] Bray NL, Pimentel H, Melsted P, Pachter L (2016). Near-optimal probabilistic RNA-seq quantification. Nat Biotechnol.

[81] Eyre DW, Davies KA, Davis G, Fawley WN, Dingle KE (2018). Two distinct patterns of Clostridium difficile diversity across Europe indicating contrasting routes of spread. Clin Infect Dis.

[82] Janežič S, Strumbelj I, Rupnik M (2011). Use of modified PCR ribotyping for direct detection of Clostridium difficile ribotypes in stool samples. J Clin Microbiol.

[83] He M, Sebaihia M, Lawley TD, Stabler RA, Dawson LF (2010). Evolutionary dynamics of Clostridium difficile over short and long time scales. Proc Natl Acad Sci U S A.

[84] Songer JG, Trinh HT, Killgore GE, Thompson AD, McDonald LC (2009). *Clostridium difficile* in retail meat products, USA, 2007. Emerg Infect Dis.

[85] Didelot X, Eyre DW, Cule M, Ip CLC, Ansari MA (2012). Microevolutionary analysis of Clostridium difficile genomes to investigate transmission. Genome Biol.

[86] Dridi L, Tankovic J, Petit J-C (2004). CdeA of *Clostridium difficile*, a new multidrug efflux transporter of the mate family. Microb Drug Resist.

[87] Farrow KA, Lyras D, Rood JI (2001). Genomic analysis of the erythromycin resistance element Tn5398 from *Clostridium difficile*. Microbiology.

[88] Spigaglia P, Carucci V, Barbanti F, Mastrantonio P, determinants E (2005). ErmB determinants and Tn916-Like elements in clinical isolates of *Clostridium difficile*. Antimicrob Agents Chemother.

[89] Sebaihia M, Wren BW, Mullany P, Fairweather NF, Minton N (2006). The multidrug-resistant human pathogen *Clostridium difficile* has a highly mobile, mosaic genome. Nat Genet.

[90] Coque TM, Singh KV, Weinstock GM, Murray BE (1999). Characterization of dihydrofolate reductase genes from trimethoprim-susceptible and trimethoprim-resistant strains of *Enterococcus faecalis*. Antimicrob Agents Chemother.

[91] Bergmann R, van der Linden M, Chhatwal GS, Nitsche-Schmitz DP (2014). Factors that cause trimethoprim resistance in *Streptococcus pyogenes*. Antimicrob Agents Chemother.

[92] Boyd DA, Du T, Hizon R, Kaplen B, Murphy T (2006). VanG-type vancomycin-resistant *Enterococcus faecalis* strains isolated in Canada. Antimicrob Agents Chemother.

[93] Wasels F, Monot M, Spigaglia P, Barbanti F, Ma L (2014). Inter- and intraspecies transfer of a *Clostridium difficile* conjugative transposon conferring resistance to MLSB. Microb Drug Resist.

[94] Riedel T, Wetzel D, Hofmann JD, Plorin SPEO, Dannheim H (2017). High metabolic versatility of different toxigenic and non-toxigenic *Clostridioides difficile* isolates. Int J Med Microbiol.

[95] Goudarzi M, Goudarzi H, Alebouyeh M, Azimi Rad M, Shayegan Mehr FS (2013). Antimicrobial susceptibility of *Clostridium difficile* clinical isolates in Iran. Iranian Red Crescent Medical Journal.

[96] Chow VCY, Kwong TNY, So EWM, Ho YII, Wong SH (2017). Surveillance of antibiotic resistance among common *Clostridium difficile* ribotypes in Hong Kong. Sci Rep.

[97] Knetsch CW, Hensgens MPM, Harmanus C, van der Bijl MW, Savelkoul PHM (2011). Genetic markers for *Clostridium difficile* lineages linked to hypervirulence. Microbiology.

[98] Kansau I, Barketi-Klai A, Monot M, Hoys S, Dupuy B (2016). Deciphering adaptation strategies of the epidemic *Clostridium difficile* 027 strain during infection through in vivo transcriptional analysis. PLoS One.

[99] Jhung MA, Thompson AD, Killgore GE, Zukowski WE, Songer G (2008). Toxinotype V *Clostridium difficile* in humans and food animals. Emerg Infect Dis.

[100] Goorhuis A, Bakker D, Corver J, Debast SB, Harmanus C (2008). Emergence of *Clostridium difficile* infection due to a new hypervirulent strain, polymerase chain reaction ribotype 078. Clin Infect Dis.

[101] Bauer MP, Notermans DW, van Benthem BHB, Brazier JS, Wilcox MH (2011). *Clostridium difficile* infection in Europe: a hospital-based survey. The Lancet.

[102] Keel K, Brazier JS, Post KW, Weese S, Songer JG (2007). Prevalence of PCR ribotypes among *Clostridium difficile* isolates from pigs, calves, and other species. J Clin Microbiol.

[103] Burt SA, Siemeling L, Kuijper EJ, Lipman LJA (2012). Vermin on pig farms are vectors for *Clostridium difficile* PCR ribotypes 078 and 045. Vet Microbiol.

[104] Weese JS (2010). *Clostridium difficile* in food-innocent bystander or serious threat?. Clin Microbiol Infect.

[105] Stone NE, Nunnally AE, Jimenez V, Cope EK, Sahl JW (2019). Domestic canines do not display evidence of gut microbial dysbiosis in the presence of *Clostridioides (Clostridium) difficile*, despite cellular susceptibility to its toxins. Anaerobe.

[106] Galinier A, Deutscher J (2017). Sophisticated regulation of transcriptional factors by the bacterial phosphoenolpyruvate: sugar phosphotransferase system. J Mol Biol.

[107] Chen J-W, Scaria J, Mao C, Sobral B, Zhang S (2013). Proteomic comparison of historic and recently emerged hypervirulent *Clostridium difficile* strains. J Proteome Res.

[108] Scaria J, Mao C, Chen J-W, McDonough SP, Sobral B (2013). Differential stress transcriptome landscape of historic and recently emerged hypervirulent strains of *Clostridium difficile* strains determined using RNA-seq. PLoS One.

[109] Xiao M, Kong F, Jin P, Wang Q, Xiao K (2012). Comparison of two capillary gel electrophoresis systems for *Clostridium difficile* ribotyping, using a panel of ribotype 027 isolates and whole-genome sequences as a reference standard. J Clin Microbiol.

[110] Valiente E, Cairns MD, Wren BW (2014). The *Clostridium difficile* PCR ribotype 027 lineage: a pathogen on the move. Clin Microbiol Infect.

[111] Pearson T, Busch JD, Ravel J, Read TD, Rhoton SD (2004). Phylogenetic discovery bias in *Bacillus anthracis* using single-nucleotide polymorphisms from whole-genome sequencing. Proc Natl Acad Sci U S A.

[112] McLure A, Clements ACA, Kirk M, Glass K (2017). Healthcare-associated *Clostridium difficile* infections are sustained by disease from the community. Bull Math Biol.

[113] Leffler DA, Lamont JT (2015). *Clostridium difficile* infection. N Engl J Med.

[114] Bakker D, Corver J, Harmanus C, Goorhuis A, Keessen EC (2010). Relatedness of human and animal *Clostridium difficile* PCR ribotype 078 isolates determined on the basis of multilocus variable-number tandem-repeat analysis and tetracycline resistance. J Clin Microbiol.

[115] Knetsch CW, Kumar N, Forster SC, Connor TR, Browne HP (2018). Zoonotic transfer of *Clostridium difficile* harboring antimicrobial resistance between farm animals and humans. J Clin Microbiol.

[116] Dingle KE (2018). A role for tetracycline selection in the evolution of *Clostridium difficile* PCR-ribotype 078. bioRxiv.

[117] Xu C, Weese JS, Flemming C, Odumeru J, Warriner K (2014). Fate of *Clostridium difficile* during wastewater treatment and incidence in Southern Ontario watersheds. Journal of Applied Microbiology.

[118] Moran NA (2002). Microbial minimalism: genome reduction in bacterial pathogens. Cell.

[119] Stinear TP, Seemann T, Pidot S, Frigui W, Reysset G (2007). Reductive evolution and niche adaptation inferred from the genome of *Mycobacterium ulcerans*, the causative agent of Buruli ulcer. Genome Res.

[120] Drudy D, Goorhuis B, Bakker D, Kyne L, van den Berg R (2008). Clindamycin-resistant clone of *Clostridium difficile* PCR ribotype 027, Europe. Emerg Infect Dis.

[121] Sahl JW, Steinsland H, Redman JC, Angiuoli SV, Nataro JP (2011). A comparative genomic analysis of diverse clonal types of enterotoxigenic *Escherichia coli* reveals pathovar-specific conservation. Infect Immun.

[122] Williamson CHD, Sanchez A, Gutman J, Sahl JW (2016). Bacterial genome reduction as a result of short read sequence data. biorXiv.

