# Supplementary material for: A global to local genomics analysis of Clostridioides difficile ST1/RT027 identifies cryptic transmission events in a northern Arizona healthcare network
Source: Microb Genom. 2019 May 20;5(7):e000271. doi: 10.1099/mgen.0.000271 (PMC6700662; doi:10.1099/mgen.0.000271)
Supplement: Supplementary File 1 [file mgen-5-271-s001.pdf]

## Supplementary Figures

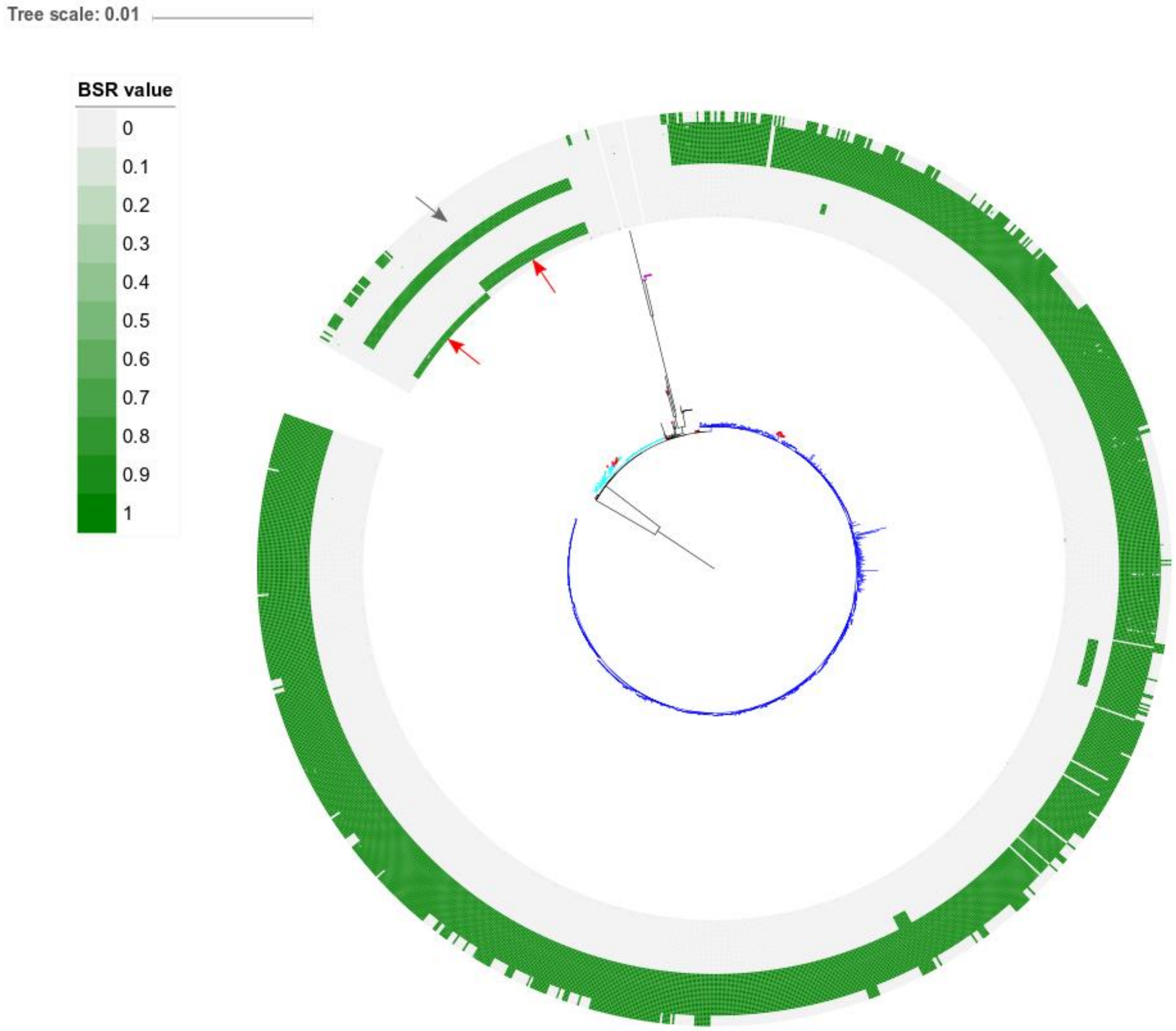


**Figure S1**. Phylogeny of ST1 genomes paired with a heatmap indicating BSR values of a subset of proteins that are differentially conserved throughout ST1. Red arrows indicate examples of lineage-specific genes/proteins, and the gray arrow indicates an example of genes/proteins that are absent in a specific lineage.

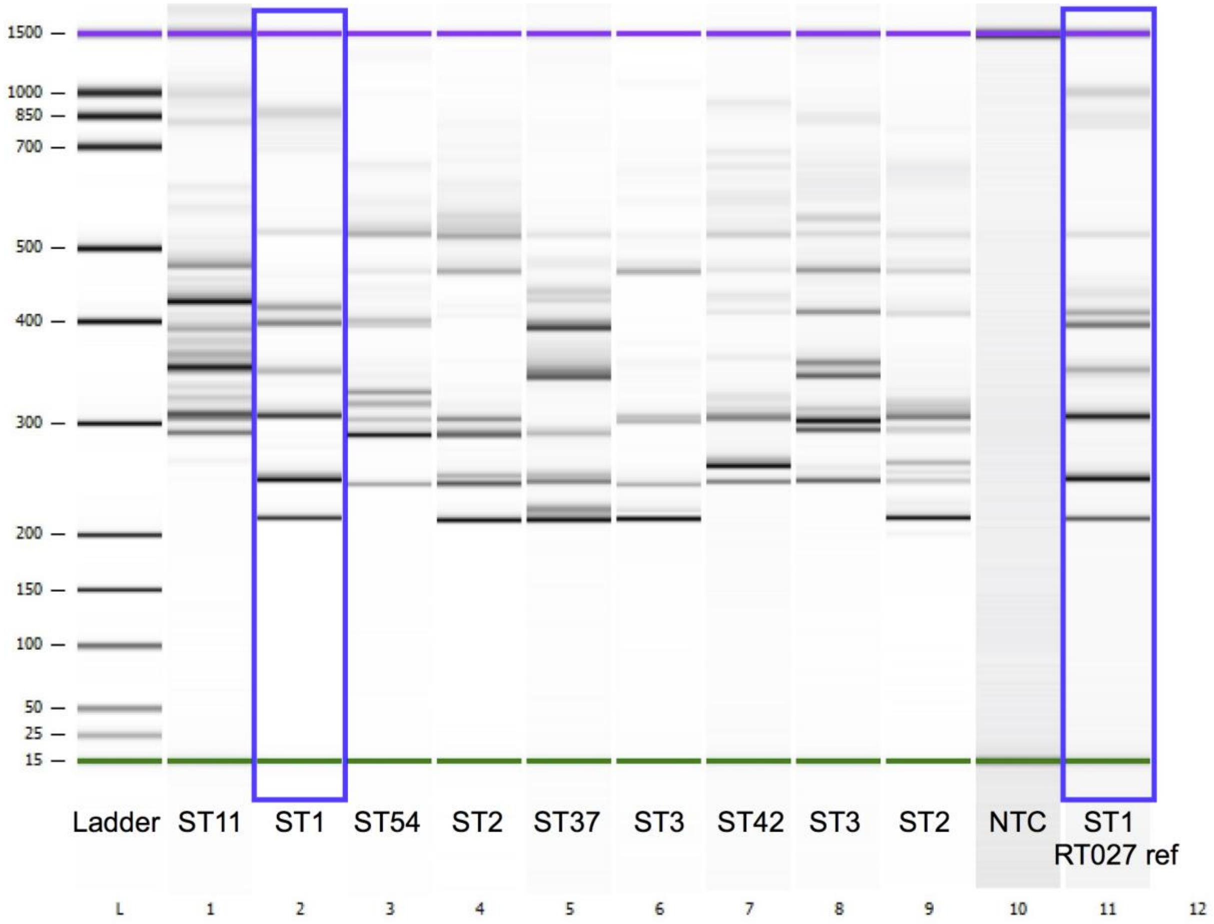


**Figure S2**: Bioanalyzer gel image for PCR ribotyping of *C. difficile* isolates. Lanes identified with blue boxes include ST1 isolates. The ST1 isolates have similar PCR ribotyping profiles, which are comparable to *in silico* ribotyping results (see main text). ST3 contains multiple ribotypes and does not form a monophyletic clade based upon a core genome SNP phylogeny (see Figure 1); ST3 isolates tested here present multiple PCR ribotyping profiles.

**Supplementary Tables**

Table S1: External genomes analyzed in this study (Available as supplementary file)

Table S2: NASP matrix of SNPs for global collection of *C. difficile* (Available as supplementary file)

Table S3: NASP matrix of SNPs for *C. difficile* ST1 genomes (Available as supplementary file)

Table S4: SNPs for timing analysis (Available as supplementary file)

Table S5: Assessment of statistical fits for clock and demographic model combination (Available as supplementary file)

Table S6: Previously described genomic regions associated with virulence in *C. difficile* ST1 (Available as supplementary file)

Table S7: Ribotype region information for three complete *C. difficile* ST1 genomes (Available as supplementary file)

Table S8: Results for screening genomes for antimicrobial resistance markers (CARD database) with LB-BSR (Available as supplementary file)

Table S9: Summary of transposon presence in ST1 genomes (Available as supplementary file)

Table S10: Summary of GyrA and GyrB mutations in *C. difficile* (Available as supplementary file)

Table S11: Results for antimicrobial resistance screening with Etests (Available as supplementary file)

Table S12: The strict core genome identified with LS-BSR for *C. difficile* ST1 genomes (Available as supplementary file)

Table S13: CDSs identified as 'gained' and 'lost' within *C. difficile* ST1 (Available as supplementary file)

Table S14: CDSs lost in two northern Arizona isolates from the same patient (Available as supplementary file)

Table S15: Results for screening genomes for sequences or markers associated with *C. difficile* virulence (Available as supplementary file)

Table S16. *In silico* ribotyping threshold summary (Available as supplementary file)

Table S17: *In silico* ribotyping results for all analyzed genomes (Available as supplementary file)

Table S18: Comparison of *in silico* and PCR ribotyping results (Available as supplementary file)
